# Cardiometabolic risk phenotypes and chronic kidney disease incidence in older adults: a nationwide longitudinal cohort study

**DOI:** 10.1186/s12889-025-23868-w

**Published:** 2025-07-29

**Authors:** Zhihe Zeng, Zhaoyang Xiao

**Affiliations:** Department of Anesthesiology, The Second Affiliated Hospital of Dalian Medical University, Dalian, 116027 China

**Keywords:** Cardiometabolic disease, Latent class analysis, Chronic kidney disease, Cardiometabolic phenotype, Sensitivity analysis

## Abstract

**Background:**

There is mixed evidence for an association between cardiometabolic risk factors and chronic kidney disease risk (CKD). This study aimed to determine whether different latent classes of cardiometabolic conditions were associated with chronic kidney disease risk.

**Method:**

Data from 7,195 participants in the China Health and Retirement Longitudinal Study (CHARLS) were analyzed. Latent class analysis was performed using data on obesity, high-density lipoprotein cholesterol, triglyceride, hypertension, diabetes, arthritis or rheumatism, and systemic inflammatory conditions and heart disease. Confounder-adjusted multiple logistic regressions were conducted to estimate CKD incidence by cardiometabolic latent classes. Sensitivity analyses were performed across cross-sectional and longitudinal samples, as well as derivation and validation cohorts.

**Results:**

Three cardiometabolic classes were identified: relatively healthy cardiometabolic (RHC) phenotype, metabolic syndrome (MetS) phenotype, and cardiovascular disease (CVD) phenotype, which accounted for 66.2%, 19.9%, and 13.8%, respectively. The incidence of CKD was 12.7% in the CVD group, 9.4% in the MetS group, and 5.9% in the RHC group. After adjusting for confounding factors, it was found that the metabolic syndrome type had a 54% increased risk of newly diagnosed CKD compared to the healthy heart type (OR = 1.54, 95% CI: 1.22–1.93), while the cardiovascular type increased by 104% (OR = 2.04, 95% CI: 1.61–2.57). Sensitivity analyses showed high consistency (> 90%) in class assignments, confirming model robustness.

**Conclusion:**

Different cardiometabolic phenotypes are associated with an increased risk of new-onset CKD. Gender and age are important factors influencing the strength of this association. Phenotypic classification may improve CKD risk stratification and guide early prevention efforts.

**Supplementary Information:**

The online version contains supplementary material available at 10.1186/s12889-025-23868-w.

## Introduction

Chronic kidney disease (CKD) is a global health issue affecting millions of people worldwide, reducing their quality of life and life expectancy [1]. As the disease progresses, CKD not only increases the risk of cardiovascular disease but may also lead to renal failure, necessitating lifelong dialysis or kidney transplantation. The development of CKD has been closely linked to various metabolic abnormalities, including established risk factors like obesity, diabetes, and hypertension [2].

Cardiometabolic syndrome (CMS), a cluster of risk factors including obesity, diabetes, and hypertension, has garnered significant attention due to its potential link to CKD [3]. These factors not only independently influence CKD risk, but also interact within the CMS framework, leading to substantial heterogeneity in its clinical presentations [4, 5]. This heterogeneity complicates studying the CMS-CKD relationship when treating CMS as a single entity [6–8]. This underscores the importance of in-depth research on the association between specific CMS phenotypes and CKD. Recent research on CMS has moved beyond traditional disease combination models by subdividing it into distinct clinical phenotypes based on the varying combinations of risk factors observed in patients [4, 5]. In this respect, some studies have classified CMS into phenotypes such as metabolic syndrome, hypertension, and obesity, aiming to explore the relationship between these phenotypes and other diseases [5]. However, research on the association between these different phenotypes and the development of CKD remains limited. Particularly within the complex interplay between CMS and CKD, research on how specific CMS phenotypes influence the risk of new-onset CKD requires further investigation.

The introduction of the cardiovascular-kidney-metabolic (CKM) syndrome concept provides a new framework for understanding the complex interplay between cardiometabolic syndrome and CKD [9–11]. This concept challenges the traditional view of heart and kidney diseases as independent processes, revealing their potential close connections. However, the detailed mechanisms by which different metabolic syndrome phenotypes, independently or collectively, influence CKD development remain largely unexplored. Therefore, through a retrospective analysis of a specific population, we hypothesize that certain phenotypes of cardiometabolic syndrome significantly elevate the risk of CKD. Validating this hypothesis will not only deepen our understanding of the heart-kidney interaction under metabolic abnormalities, but also provide empirical support for the CKM syndrome concept. This, in turn, could facilitate the development of targeted preventive measures and treatment strategies. Overall, this study holds significant value for advancing scientific knowledge in cardiovascular and renal health, providing a theoretical and practical foundation for applying the new CKM syndrome concept.

## Methods

### Design and sample

The China Health and Retirement Longitudinal Study (CHARLS), launched in 2011, recruited a nationally representative sample of 17,708 participants aged 45 and older across 28 provinces in China. Multistage sampling was employed, and data collection utilized one-on-one interviews and questionnaires to gather demographic, lifestyle, and health information. Follow-up assessments have been conducted every two years since baseline, with efforts to re-interview the same participants for longitudinal tracking. Although refreshment samples have occasionally been added in later waves to maintain national representativeness, the CHARLS cohort is designed to follow the same individuals over time [12].

**Fig. 1:**
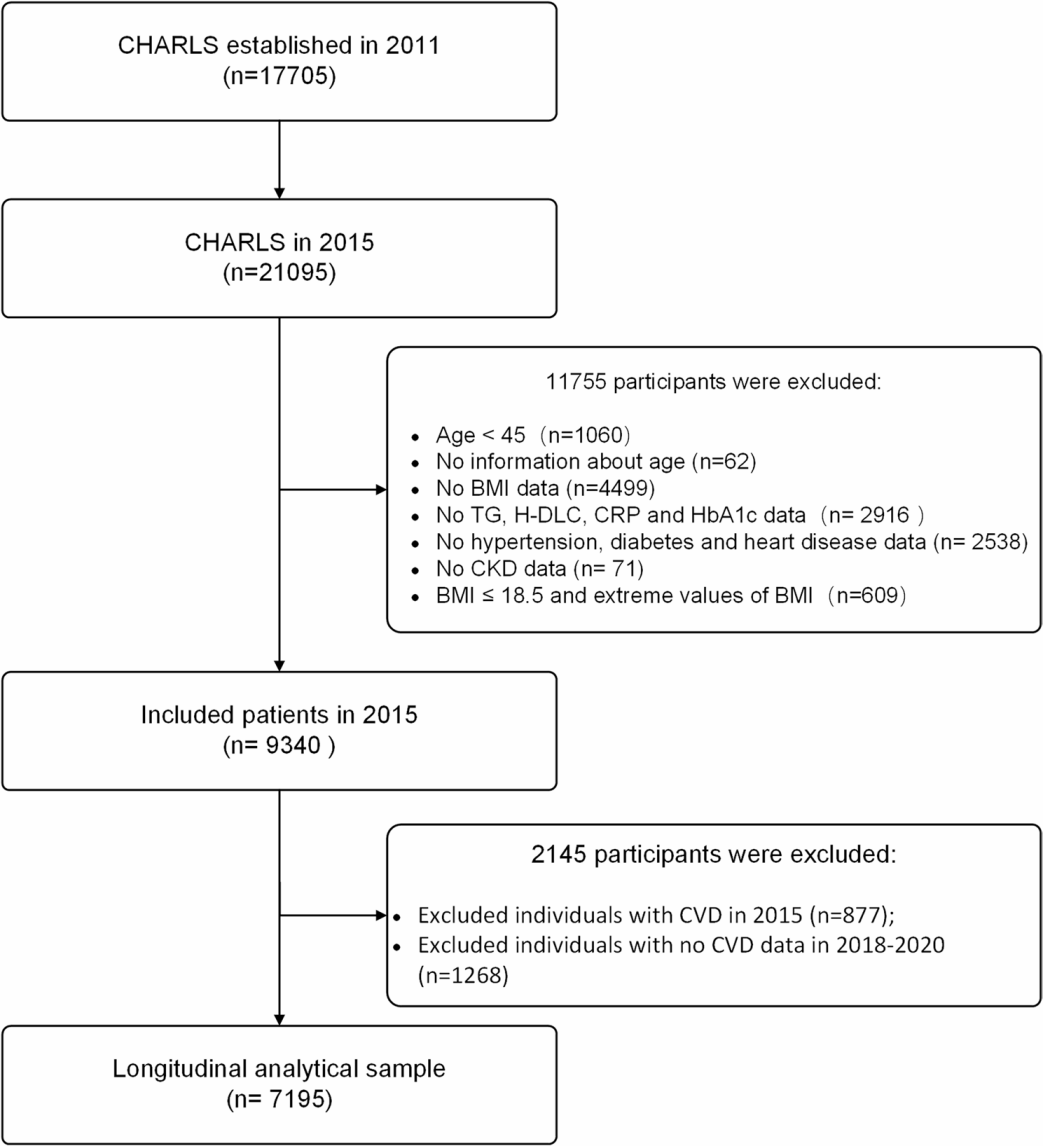
Flow diagram for participants included in the study

In this study, we retrospectively analyzed data from the China Health and Retirement Longitudinal Study (CHARLS), selecting the 2015 wave as the analytic baseline because it was the first to include systematic collection of blood biomarkers such as triglycerides and glycated hemoglobin (HbA1c). A total of 21,095 participants from the 2015 baseline were initially considered for inclusion. We excluded individuals with chronic kidney disease (CKD) at baseline, those missing biomarker data, and those without complete CKD follow-up information through 2020. Based on the study protocol, participants were sequentially excluded for the following reasons: age < 45 years (*n* = 1,060), missing age information (*n* = 62), missing body mass index (BMI) data (*n* = 4,499), incomplete cardiovascular-metabolic data—including triglycerides (TG), high-density lipoprotein cholesterol (HDL-C), C-reactive protein (CRP), and glycated hemoglobin (HbA1c) (*n* = 2,916)—and missing medical history data on hypertension, diabetes, or heart disease (*n* = 2,538). Additionally, individuals with missing information on chronic kidney disease (CKD) status (*n* = 71) or with underweight (BMI ≤ 18.5) or extreme BMI values (*n* = 609) were excluded. After these exclusions, 9,340 participants with complete cardiovascular-metabolic data at baseline were retained for further analysis. Participants with prevalent CKD at baseline (*n* = 877) or without follow-up CKD data between 2018 and 2020 (*n* = 1,268) were subsequently excluded, yielding a final cohort of 7,195 individuals included in the study. The selection process is detailed in Fig. 1. All participants provided written informed consent and the ethics review committee of Peking University approved the CHARLS protocol.

### Cardiometabolic conditions

The construction of latent classes involved the consideration of eight cardiometabolic conditions, selected with reference to previous latent class analyses [4, 5] of cardiometabolic phenotypes: obesity, reduced plasma high-density lipoprotein cholesterol (HDL-C), elevated plasma triglyceride (TG), hypertension, diabetes, arthritis or rheumatism, systemic inflammatory conditions (indicated by raised C-reactive protein), and heart disease, all derived from the baseline assessment and defined according to guidelines [13–16] (Supplementary Table 1). Each of the eight cardiometabolic indicators was considered as a separate binary variable based on the presence or absence of an abnormal indicator.

### Chronic kidney disease

In this study, CKD status was determined using an integrated approach. This approach combined participant-reported physician diagnoses of kidney disease (excluding tumors or cancers) with quantitative assessments of Glomerular Filtration Rate (GFR) using estimated GFR (eGFR) values. This method was applied at three time points: baseline (2015) and two follow-up surveys in 2018 and 2020. Participants were asked if they had ever been diagnosed with kidney disease by a doctor (including reports from proxies). Individuals with a positive response and an eGFR below 60 mL/min/1.73 m² were classified as CKD patients. The CKD-EPI formula was used to calculate GFR following established guidelines [17]:

$$\begin{aligned} GFR=&141\;\ast\;\min{\;\left(Scr/\kappa,1\right)}^{\;\mathrm\alpha\;}\ast\;\max{\;\left(Scr/\kappa,1\right)}^{-1.209}\;\\&\ast\;{0.993}^{Age}\;\ast\;1.018\;\left[if\mathit\;female\right],\;\; \end{aligned}$$

where Scr is serum creatinine, κ is 0.7 for females and 0.9 for males, α is −0.329 for females and 0.411 for males, min indicates the minimum, and max indicates maximum.

### Covariates

This study comprehensively assessed covariates associated with CKD, including sociodemographic factors, lifestyle habits, co-morbidities, and key blood indicators. Sociodemographic factors included age, gender, education level, marital status, and living environment. Lifestyle habits were assessed through nighttime sleep duration and inquiries about smoking and drinking habits. Additionally, the study screened for important co-morbid histories such as cancer, chronic lung disease, and stroke. Blood analyses were conducted to measure white blood cells, hemoglobin, and serum creatinine, providing a comprehensive assessment of overall health status and potential correlations with CKD (Supplementary Table 2).

### Latent class construction

Latent class analysis (LCA) is a statistical technique used to explore unobserved groupings (latent classes) within a population by identifying individuals who share similar patterns across a set of pre-selected measurable conditions related to cardiometabolic health [18]. Latent class analysis addresses the challenge of identifying unobserved subgroups within a population by leveraging the concept of posterior probability. In LCA, individuals are assigned to latent classes based on the highest probability of their observed characteristics aligning with the underlying profile of a particular class. This probabilistic approach ensures that each class is independent and distinct [19]. Rather than requiring every member of a class to perfectly match a specific profile, LCA focuses on the overall probability distribution of observed characteristics within each class. This simplifies the process of defining and identifying latent classes. A core aspect of LCA is the iterative process of determining the optimal number of classes. Instead of pre-specifying the number of subgroups, LCA evaluates various configurations using information criteria like Akaike Information Criterion (AIC) and Adjusted Bayesian Information Criterion (BIC) to identify the model that best explains the data [20]. These criteria help identify the model that best balanced goodness-of-fit and model complexity. Once the optimal number of classes was determined, individuals were assigned to a specific latent class based on the highest probability of belonging to that class. Classes with an average posterior probability exceeding 0.70 were considered well-defined, indicating a strong likelihood of membership for individuals within that class [19]. Latent class characteristics were defined by Item response probabilities, interpreted with a conditional probability of 0.5, consistent with previous research [21].

### Statistical analysis

Baseline characteristics are presented as percentages for categorical variables and as mean ± standard deviation (if normally distributed) or median and interquartile range (if skewed) for continuous variables. Continuous variables were compared using ANOVA or Kruskal-Wallis tests, depending on normality. Categorical variables were compared using chi-square tests. Multivariate logistic regression models were employed to analyze the association between cardiometabolic diseases and CKD. These models included demographic factors, lifestyle habits, additional comorbidities, and blood test data as covariates. Subgroup analyses were performed to assess potential variations in the association by gender, age, and sleep duration. Odds ratios (OR) and their corresponding 95% confidence intervals (CI) were calculated [22] for all analyses. A two-tailed *p*-value of less than 0.05 was considered statistically significant. This study adhered to the Strengthening the Reporting of Observational Studies in Epidemiology (STROBE) reporting guidelines [23]. All statistical analyses were conducted using R software version 4.2.0 (https://www.r-project.org, The R Foundation).

### Derivation/validation dataset construction and sensitivity analyses

**Fig. 2:**
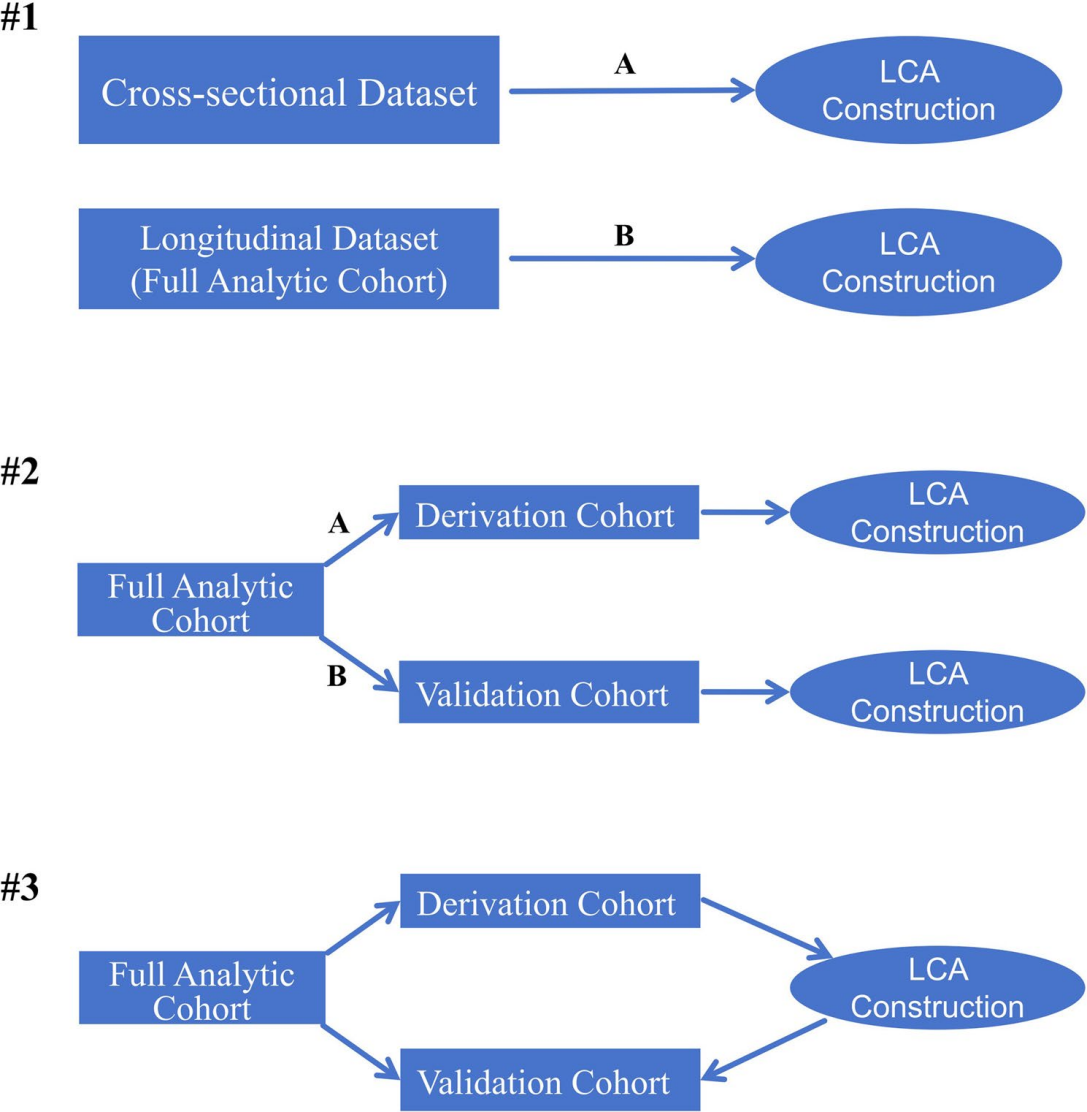
Visualizations of latent class analyses validations conducted

Given that latent class analysis (LCA) is essentially an unsupervised learning approach, the validity and reliability of the resulting class labels require additional evaluation. To better gauge the reproducibility and generalizability of our labelling strategy, we conducted a series of sensitivity analyses using derivation and validation subgroupings of the full analytic cohort (Fig. 2; Supplementary Appendix 2). First, we independently constructed a three-class LCA model using individuals from the 2015 baseline dataset who had complete cardiometabolic data (*n* = 9,340), and compared this model with the one derived from the longitudinal analytic sample utilized in the primary analysis (*n* = 7,195) to examine consistency in latent class structure (Supplementary Appendix 2: #1). Second, we randomly divided the full analytic cohort (longitudinal analytic sample, *n* = 7,195) into derivation (70%, *n* = 5,036) and validation (30%, *n* = 2,159) cohort. LCA was conducted independently within the derivation and validation cohorts. Class assignments and posterior probabilities were compared with those derived from the full cohort model to assess classification consistency and structural stability (Supplementary Appendix 2: #2). Third, latent class construction was run for the 3-class model within only those in the derivation cohort. Model coefficients were then hand-coded into those in the validation cohort, and the latent class with the highest posterior predicted value was assigned for class membership. Posterior probability of class membership was then compared with that from the latent classes constructed within the full analytic cohort (Supplementary Appendix 2: #3).

## Results

### Characteristics of latent classes

**Fig. 3:**
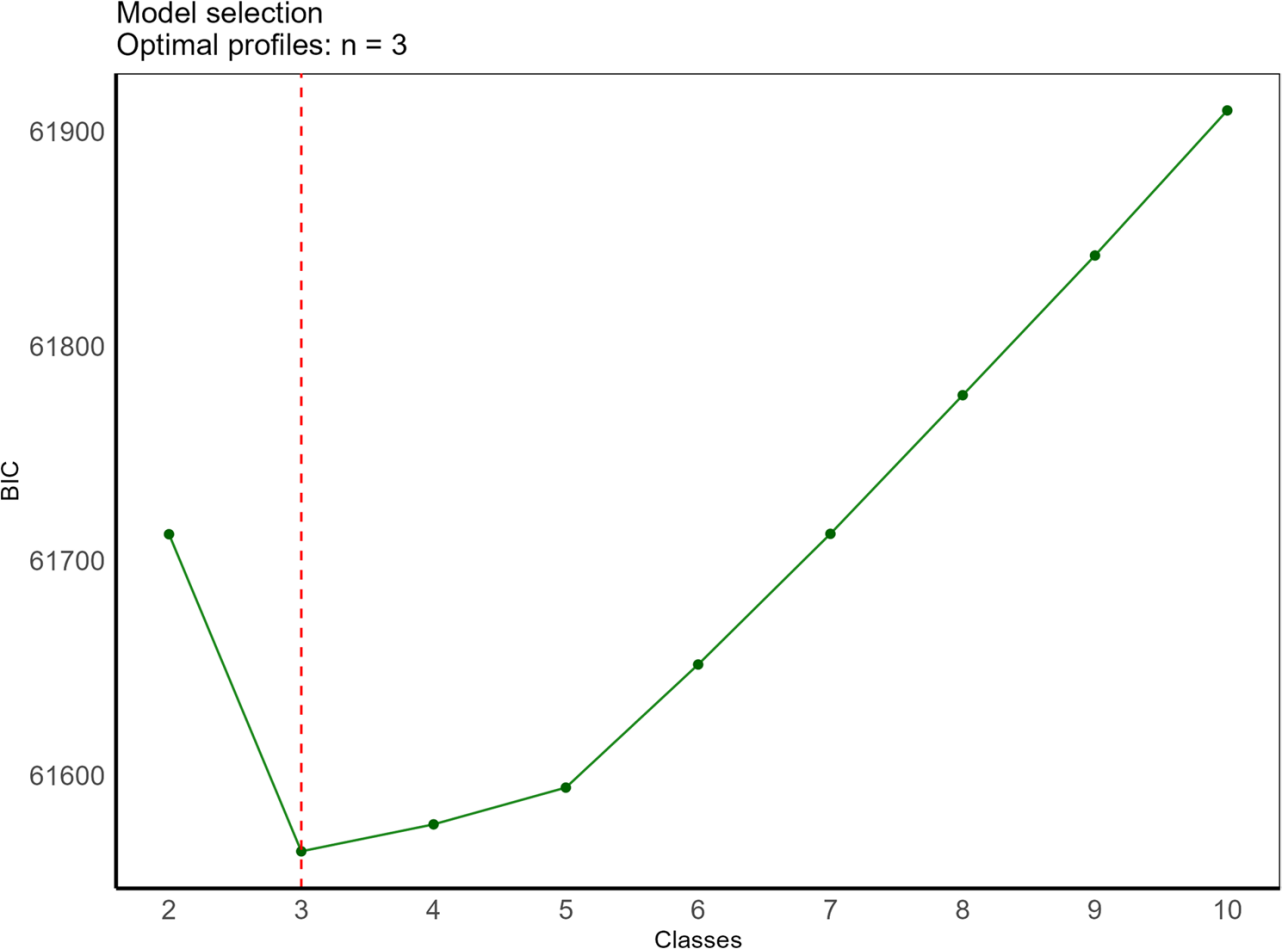
Determining the Optimal Number of Cardiometabolic Phenotypes with Latent Profile Analysis. This figure depicts the descending Bayesian information criterion (BIC) values as the number of latent profiles in the analysis increases from 2 to 10. The elbow point at 3 latent profiles (*n* = 3) suggests that a three-profile model is the most optimal solution for this dataset

**Fig. 4:**
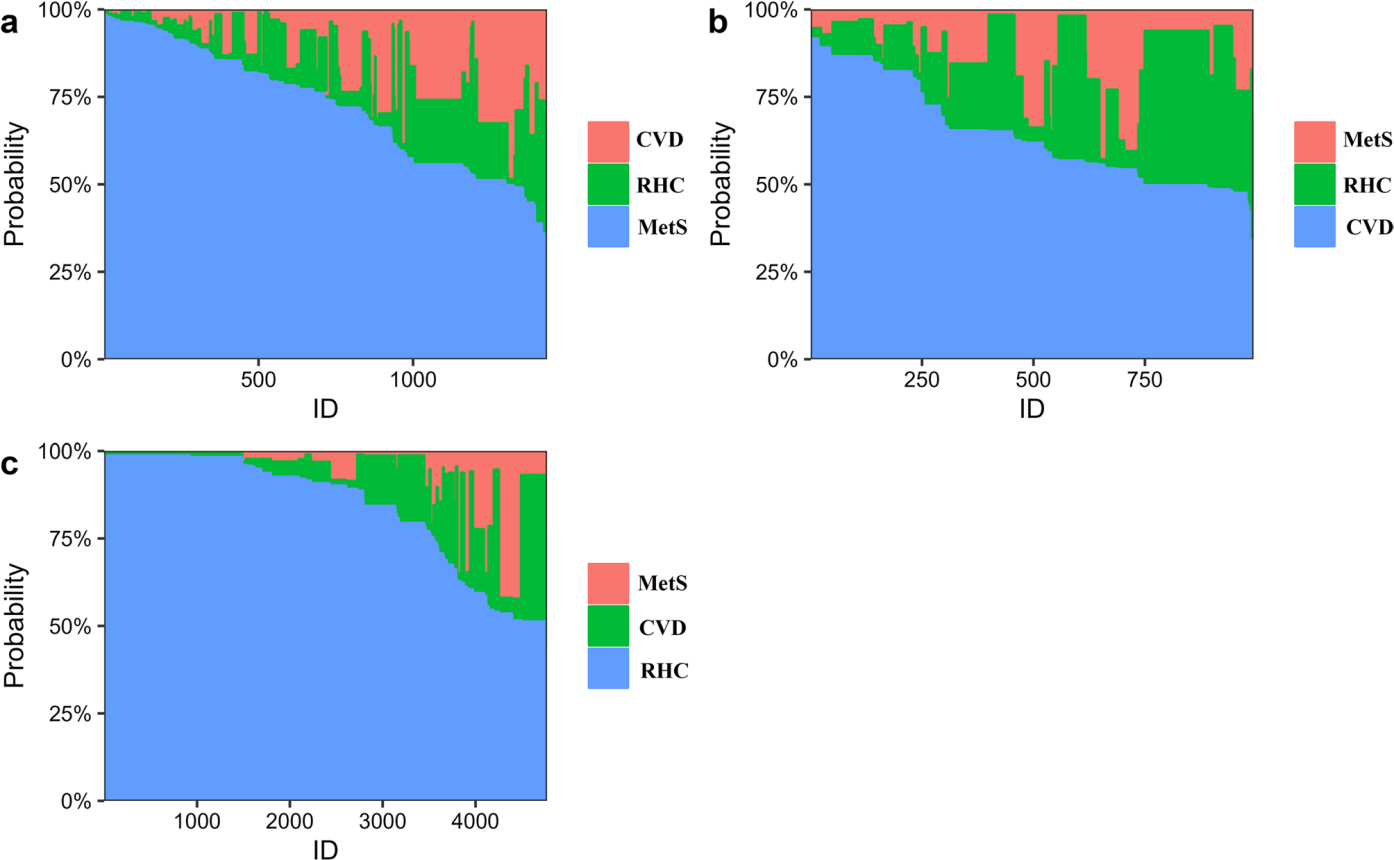
Stacked bar chart of individual-level predicted probabilities for phenotype class membership. The full probability distributions for everyone assigned to the MetS phenotype (**a**), the CVD phenotype (**b**), and the RHC phenotype (**c**). The horizontal axis represents all individuals who have been assigned to a specific phenotype. These individuals are identified by ID numbers and are arranged in a sequential order. The vertical axis corresponds to the predicted probability of class membership for each individual within a given category (MetS (**a**), CVD (**b**), RHC (**c**)). On the horizontal axis, each individual is represented by a set of stacked bars, where the height of each color segment within the bar indicates the predicted probability of belonging to the respective phenotype category

To identify significant phenotypic categorization, we explored a series of latent class models with varying numbers of classes (from 2 to 10) based on the eight cardiometabolic conditions. The BIC value indicated an optimal fit for a 3-class model (Fig. 3, Supplementary Table 3). This 3-class model provided the best balance between statistical fit and interpretability of the latent classes in a clinical context. Latent classes had generally high mean posterior probability of class membership, and were consistent across validations (Supplementary Appendix 2: sensitivity analyses for latent class analysis). Individual-level predicted category membership probabilities are shown in Fig. 4.

**Fig. 5:**
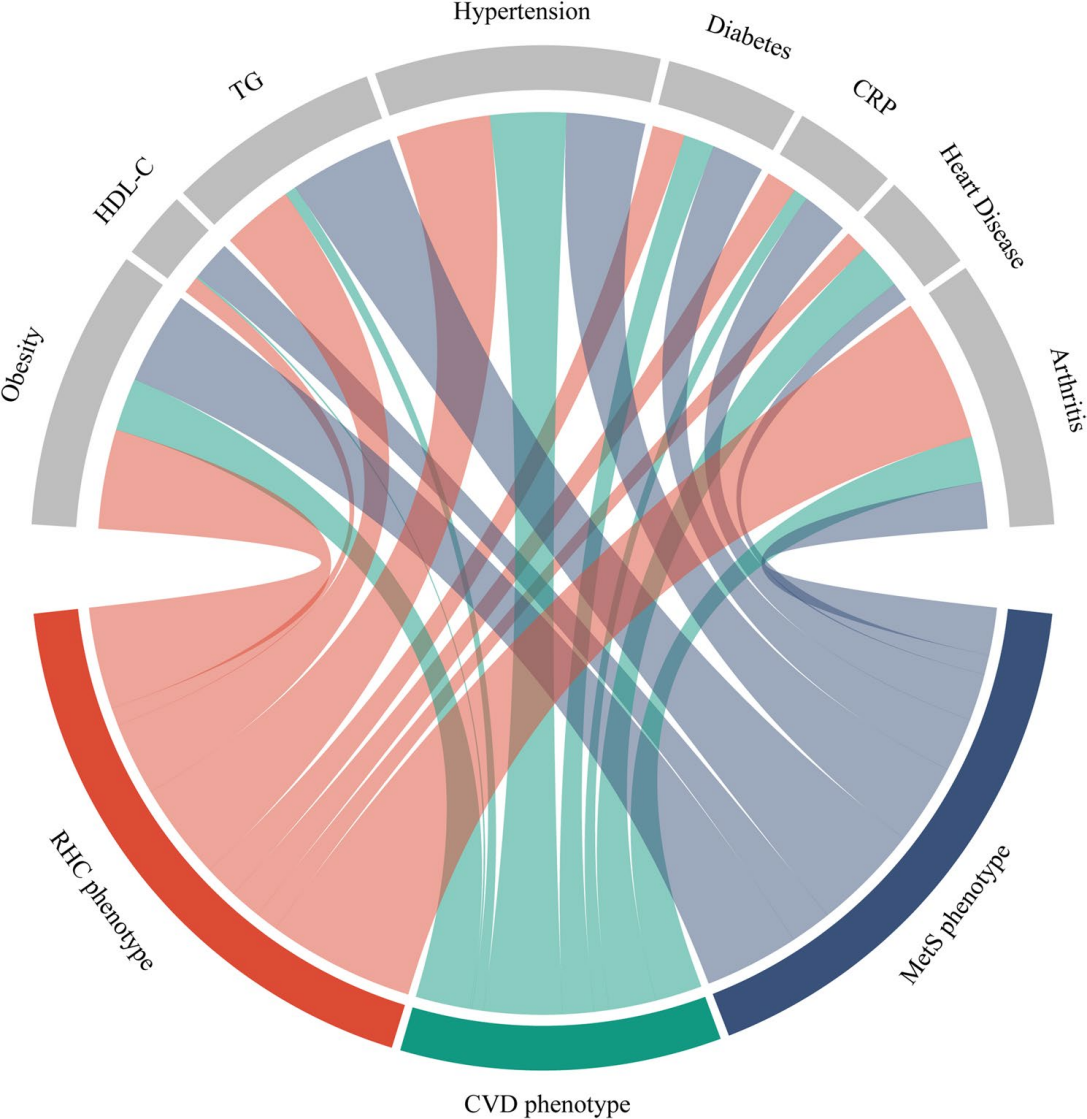
Sources of Baseline Differences between phenotypes Chord Plots illustrate the association between cardiometabolic phenotypes and clinically relevant groups of variables

Class response probabilities for each cardiometabolic risk factor are detailed in Supplementary Tables 4 and Fig. 5. Briefly, Class 1 was characterized by a high prevalence of insulin resistance (75%) and lipid abnormalities (83.2% with high triglycerides and low HDL). This profile aligned with the classic risk factor combination of metabolic syndrome, suggesting a high risk for metabolic diseases in these individuals (labeled metabolic syndrome phenotype [MetS]). Class 2 showed a relatively low prevalence of obesity and lipid issues, indicating that lipid metabolism is not a major concern in this group. However, there was a significant increase in hypertension (85.88%) and heart disease (39.07%) compared to other classes, suggesting cardiovascular disease as the predominant manifestation (labeled cardiovascular disease phenotype [CVD]). Finally, Class 3 exhibited non-significant prevalence rates across all measured conditions, particularly diabetes (9.84%) and heart disease (7.17%). Compared to the other classes with distinct metabolic abnormalities and cardiovascular risks, this class lacked typical clinical symptoms and had a lower overall disease burden (labeled relatively healthy cardiometabolic phenotype [RHC]).

### Sensitivity analyses

**Fig. 6:**
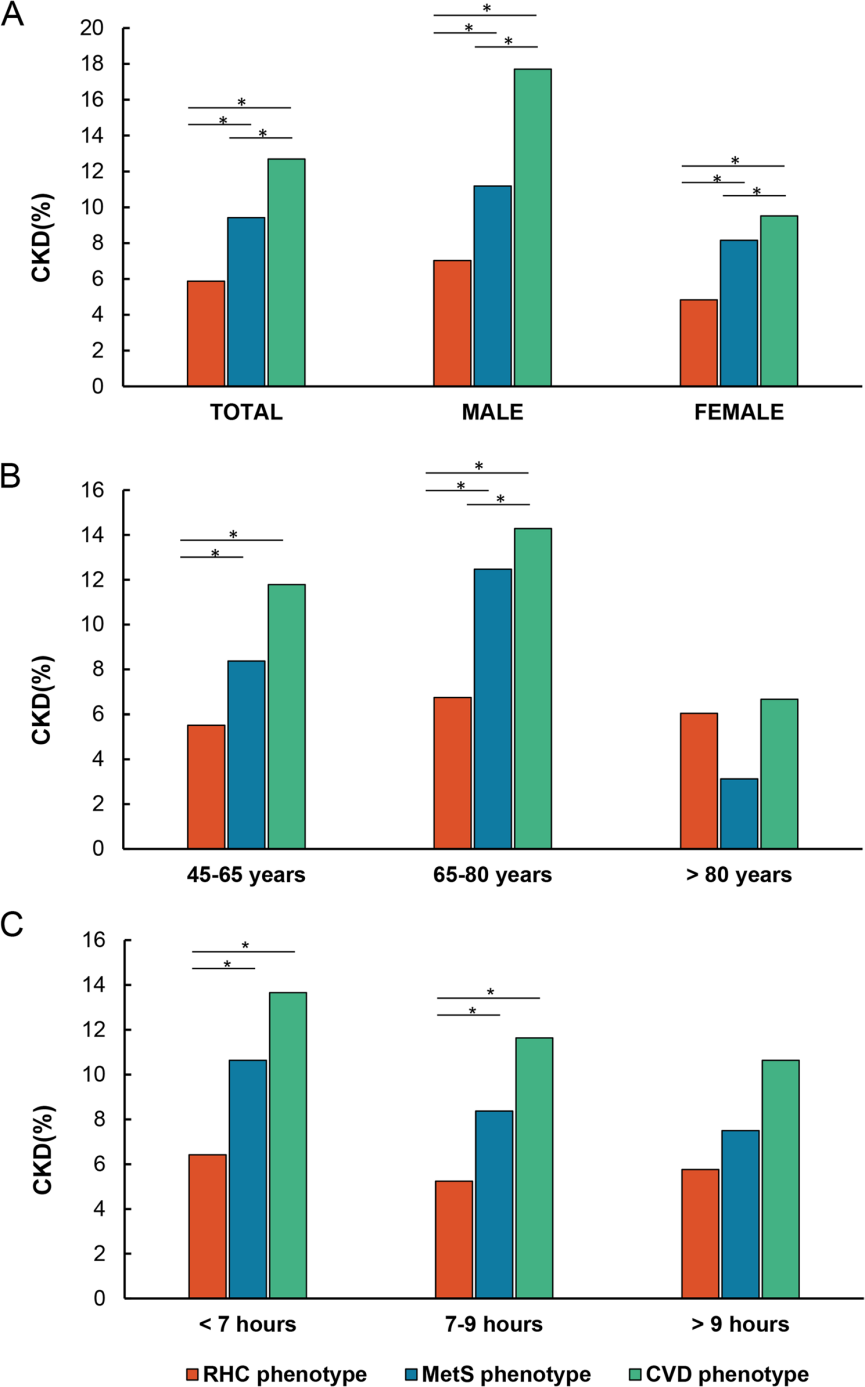
CKD risk in patients with different cardiometabolic phenotypes. **A** Total population and by gender. **B** Age groups. **C** Nighttime sleep duration. *, there is statistical difference between groups. With 3 latent classes, there are 3 distinct combinations of two classes; the Bonferroni threshold was 0.05/3 = 0.016 when comparing latent classes with each other. The *p* value of intergroup comparison was less than 0.016

We conducted three sensitivity analyses to evaluate the robustness of the LCA model. In all datasets, model selection based on the BIC consistently indicated that the 3-class solution provided the best fit. First, independent models constructed from cross-sectional (*n* = 9,340) and longitudinal datasets (full analytic cohort, *n* = 7,195) showed high classification consistency, with concordance rates of RHC 98.91%, CVD 96.58%, and MetS 97.25%. Second, models built separately in the derivation (*n* = 5,036) and validation (*n* = 2,159) cohort demonstrated strong agreement with the full analytic cohort model: RHC 97.27%, CVD 93.77%, MetS 100.00% (derivation); RHC 90.6%, CVD 97.7%, MetS 95.0% (validation). Third, applying derivation cohort parameters to the validation cohort yielded high concordance: RHC 78.41%, CVD 100.00%, MetS 98.33%, confirming the reproducibility and structural stability of the LCA solution (Table 1). Detailed results and visualizations of these sensitivity analyses, including posterior probability distributions, class-specific assignment accuracy, and predicted probability plots, are provided in Supplementary Appendix 2. This appendix includes Figs. 1, 2, 3, 4, 5, 6, 7, 8, 9, 10 and 11; Tables 1, 2, 3, 4, 5, 6, 7, 8 and 9, which correspond to each validation approach (cross-sectional vs. longitudinal, derivation/validation cohort comparisons, and coefficient transfer). These materials provide additional support for the stability and reproducibility of the LCA-derived classification structure.

**Table 1:** Comparison of latent class assignments between subsamples and the full analytic cohort in sensitivity analyses

	Full Analytic Cohort			
	Latent Class	MetS	CVD	RHC
Validation #2A (Derivation vs. Full Cohort)	MetS	1052 (100.0%)	4 (0.58%)	90 (2.73%)
	CVD	0 (0.00%)	647 (93.77%)	0 (0.00%)
	RHC	0 (0.00%)	39 (5.65%)	3204 (97.27%)
Validation #2B (Validation vs. Full Cohort)	MetS	362 (95.0%)	7 (2.3%)	0 (0.0%)
	CVD	13 (3.4%)	296 (97.7%)	138 (9.4%)
	RHC	6 (1.6%)	0 (0.0%)	1337 (90.6%)
Validation #3 (Validation vs. Full Cohort)	MetS	375 (98.33%)	0 (0.00%)	171 (11.61%)
	CVD	6 (1.57%)	303 (100.00%)	147 (9.97%)
	RHC	0 (0.00%)	0 (0.00%)	1157 (78.41%)

### Characteristics of baseline population

Table 2 summarizes the baseline characteristics of the 7,195 participants from the 2015 CHARLS according to their assigned latent class: RHC, MetS, and CVD phenotypes. The prevalence of each phenotype was 66.2% (RHC), 19.9% (MetS), and 13.8% (CVD). The prevalence of CKD observed at the end of follow-up varied across the latent classes: 12.7% in the CVD group, 9.4% in the MetS group, and 5.9% in the RHC group. The median age was highest in the CVD group (64 years) compared to the other two groups (60 years). Additionally, the CVD group had the highest proportion of participants aged 65 to 80 years and the shortest median sleep duration (6 h). The MetS group had the highest rates of obesity (81.9%) and high triglycerides (92.9%). The CVD group had significantly higher prevalence of both high blood pressure (97.4%) and heart disease (58.4%) compared to the other groups. Significant differences in co-morbidities were observed among the three groups. The CVD group had the highest prevalence of chronic lung disease and digestive diseases.

**Table 2:** The baseline characteristics of participants

Characteristics	RHC	MetS	CVD	*P* Value
Total (*n* = count)	4769(66.3)	1433(19.9)	993(13.8)	
Demographic				
Age (years, median, quartile)	60.000(53.0,66.0) a	60.000(53.0,66.0) a, b	64.000(58.0,69.0) c	< 0.001
< 65 years	3334(69.91) a	992(69.23) b	543(54.68) c	
65–80 years	1319(27.66) a	409(28.54) a, b	420(42.30) c	
> 80years	116(2.43) a	32(2.23) a, b	30(3.02) a, b	
Gender n (%)				< 0.001
Male	2248 (47.1) a	599 (41.8) b	384 (38.7) b	
Female	2521 (52.9) a	834 (58.2) b	609 (61.3) b	
Ethnicity n (%)	4508 (94.5) a	1342 (93.6) a, b	910 (91.6) b	0.002
Education n (%)				0.001
Elementary school or below	2107 (44.2) a	571 (39.8) a, b	463 (46.6) a	
Secondary school	1074 (22.5) a	337 (23.5) a, b	248 (25.0) a	
College	1082 (22.7) a	348 (24.3) a, b	188 (18.9) a	
Above	506 (10.6) a	177 (12.4) a, b	94 (9.5) a	
Marry n (%)	4203 (88.1) a	1286 (89.7) a, b	842 (84.8) c	0.001
Rural n (%)	3198 (67.1) a	815 (56.9) b	620 (62.4) c	< 0.001
Retire n (%)	514 (10.8) a	215 (15.0) b	156 (15.7) b	< 0.001
Lifestyle				
Drinking n (%)	2257 (47.3) a	600 (41.9) b	411 (41.4) b	< 0.001
Smoking n (%)	2027 (42.5) a	576 (40.2) a	370 (37.3) b	0.006
Nighttime sleep (hours, median, quartile)	6.000(5.0,8.0) a	7.000(5.0,8.0) a, b	6.000(5.0,8.0) a	0.015
< 7 h	2448(51.33) a	696(48.57) a, b	542(54.58) a	0.014
7–9 h	2078(43.57) a	657(45.85) a, b	404(40.68) a	0.041
> 9 h	243(5.10) a	80(5.58) a, b	47(4.73) a, b	0.627
Cardiometabolic Factor				
Obesity n (%)	1262 (26.5) a	1173 (81.9) b	675 (68.0) c	< 0.001
Reduced plasma HDL-C n (%)	222 (4.7) a	507 (35.4) b	26 (2.6) c	< 0.001
Elevated plasma TG n (%)	910 (19.1) a	1331 (92.9) b	147 (14.8) c	< 0.001
Hypertension n (%)	1196 (25.1) a	999 (69.7) b	967 (97.4) c	< 0.001
Diabetes n (%)	417 (8.7) a	667 (46.5) b	391 (39.4) c	< 0.001
High hsCRP n (%)	389 (8.2) a	582 (40.6) b	187 (18.8) c	< 0.001
Heart Disease n (%)	278 (5.8) a	241 (16.8) b	580 (58.4) c	< 0.001
Arthritis or Rheumatism n (%)	1819 (38.1) a	591 (41.2) a, b	582 (58.6) c	< 0.001
Additional Comorbidities				
Cancer n (%)	50 (1.0) a	18 (1.3) a	16 (1.6) ab	0.304
Chronic lung diseases n (%)	492 (10.3) a	163 (11.4) a	157 (15.8) c	< 0.001
Stroke n (%)	82 (1.7) a	64 (4.5) b	54 (5.4) b	< 0.001
Psychiatric problems n (%)	69 (1.4) a	33 (2.3) a, b	20 (2.0) a, b	0.063
Liver disease n (%)	217 (4.6) a	85 (5.9) a, b	78 (7.9) b	< 0.001
Stomach and other digestive disease n (%)	1354 (28.4) a	378 (26.4) a	345 (34.7) c	< 0.001
Asthma n (%)	176 (3.7) a	66 (4.6) a	84 (8.5) c	< 0.001
Memory related disease n (%)	75 (1.6) a	32 (2.2) a	40 (4.0) c	< 0.001
Blood Data				
WBC (10^9^/L, median, quartile)	6.000(5.0,7.0) a	6.000(5.0,7.0) b	6.000(5.0,7.0) c	< 0.001
HGB (g/L, median, quartile)	14.000(13.0,15.0) a	14.000(13.0,15.0) b	14.000(13.0,15.0) a	< 0.001
HCT	41.000(38.0,44.0) a	42.000(39.0,45.0) b	41.000(39.0,45.0) a, b	< 0.0011
Mean Corpuscular Volume	92.000(88.0,96.0) a	91.000(87.0,95.0) b	92.000(89.0,96.0) a	< 0.001
PLT (10^9^/L, median, quartile)	200.000(157.0,242.0) a	206.000(165.0,253.0) b	205.000(164.0,245.5) b	< 0.001
Scr (mg/dL, median, quartile	1.000(1.0,1.0) a	1.000(1.0,1.0) a	1.000(1.0,1.0) a, b	< 0.001
BUN (mg/L, median, quartile)	15.000(12.0,18.0) a	15.000(12.0,17.0) a	15.000(13.0,18.0) c	< 0.001
TG (mg/dL, median, quartile)	102.000(79.0,138.0) a	217.000(173.0,304.0) b	113.000(88.0,139.5) c	< 0.001
CRP (mg/L median, quartile)	1.000(1.0,2.0) a	3.000(2.0,5.0) b	2.000(1.0,3.0) c	< 0.001
SBP (mmHg, median, quartile)	122.000(112.0,134.0) a	135.000(122.0,148.0) b	141.000(129.0,152.0) c	< 0.001
DBP (mmHg, median, quartile)	73.000(66.0,80.0) a	79.000(72.0,87.0) b	80.000(73.0,88.0) b	< 0.001
HbA1c (%, median, quartile)	6.000(6.0,6.0) a	6.000(6.0,7.0) b	6.000(6.0,6.0) c	< 0.001
TC	180.000(159.0,203.0) a	190.000(166.0,217.0) b	185.000(163.0,207.0) c	< 0.001
FBG	94.000(86.0,101.0) a	105.000(94.0,130.0) b	97.000(90.0,112.0) c	< 0.001
UA (mg/dL, median, quartile)	5.000(4.0,6.0) a	5.000(4.0,6.0) b	5.000(4.0,6.0) c	< 0.001
Cystatin C (mg/L, median, quartile)	1.000(1.0,1.0) a	1.000(1.0,1.0) a	1.000(1.0,1.0) a, b	< 0.001
HDL-c (mg/dL, median, quartile)	52.000(46.0,59.0) a	42.000(37.0,49.0) b	50.000(44.0,58.0) c	< 0.001
LDL-c (mg/dl, median, quartile)	101.000(83.0,119.0) a	97.000(78.0,119.0) b	107.000(88.0,125.0) c	< 0.001
CKD n (%)	280 (5.9) a	135 (9.4) b	126 (12.7) c	< 0.001

Continuous variables are expressed as mean ± standard deviation, or as median (interquartile range). Categorical variables are expressed as frequency (percent). The small letters (a, b, c) in this table refer to comparisons between groups. There is no statistical difference between groups with the same small letters. With 3 latent classes, there are 3 distinct combinations of two classes; the Bonferroni threshold was 0.05/3 = 0.016 when comparing latent classes with each other

*BMI* Body mass index, *SBP* Systolic blood pressure, *DBP* Diastolic blood pressure, *HDL-c* High-density lipoprotein cholesterol, *TG* Triglyceride, *High hsCRP* High-sensitivity C-reactive protein levels > 3 mg/L, *WBC* White blood cell count, *HGB* Hemoglobin concentration, *HCT* Hematocrit Mean Corpuscular Volume, *PLT* Platelet, *Scr* Serum creatinine, *BUN* Blood urea nitrogen, *HbA1c* Glycated hemoglobin, *TC* Total cholesterol, *FBG* Fasting blood glucose, *UA* Uric acid, *CysC* Cystatin C, *HDL-c* High-density lipoprotein cholesterol, *LDL-c* Low-density lipoprotein cholesterol, *CKD* Chronic kidney diseases, *IQR* Interquartile range

A higher percentage of participants with new-onset CKD was observed in the MetS group (135, 9.42%) and the CVD group (126, 12.69%) compared to the RHC group (280, 5.87%) (Table 3). Among participants aged 65 to 80 years, the incidence of CKD differed significantly between the CVD and MetS groups (*p* < 0.05) (Fig. 6). In contrast, no significant difference in CKD incidence between these two phenotypes was observed in participants aged 45 to 65 years. Sleep duration was also not significantly different between the MetS and CVD groups. No statistically significant differences were observed among participants aged ≥ 80 years or those with sleep duration > 9 h.

**Table 3:** Baseline characteristics of CKD patients

CKD	RHC	MetS	CVD
TOTAL	280 (5.87) a	135 (9.42) b	126 (12.69) c
Gender			
Male	158 (7.03) a	67 (11.18) b	68 (17.71) c
Female	122 (4.84) a	68 (8.15) b	58 (9.52) c
Age (years)			
< 65 years	184 (5.52) a	83 (8.37) b	64 (11.79) b
65–80 years	89 (6.75) a	51 (12.47) b	60 (14.29) c
> 80years	7 (6.03) a	1 (3.13) a, b	2 (6.67) a, b
Nighttime sleep (hours)			
< 7 h	157 (6.31) a	74 (10.63) b	74 (13.65) b
7–9 h	109 (5.25) a	55 (8.37) b	47 (11.63) b
> 9 h	14 (5.76) a	6 (7.5) a, b	5 (10.64) a, b

### Association between cardiometabolic syndrome phenotype and new-onset CKD

Following a five-year follow-up, a total of 541 participants (7.5%) developed new-onset CKD. Analyzing the results in Table 4, we explored the associations between the three distinct cardiometabolic phenotypes and the incidence of CKD in our study. After adjusting for potential confounding factors, we observed a significant increase in the risk of new-onset CKD compared to the RHC phenotype class. The MetS group exhibited a 54% increased risk (odds ratio [OR] 1.54, 95% confidence interval [CI] 1.22–1.93), while the CVD group showed a 104% increased risk (OR 2.04, 95% CI 1.61–2.57). To further evaluate the population-level impact of each latent cardiometabolic phenotype, we estimated the population attributable risk (PAR) for incident CKD using adjusted odds ratios (ORs) and class prevalence. As shown in Supplementary Table 5, the MetS class accounted for 9.7% of the total CKD risk, while the CVD class contributed 12.6%, together explaining approximately 22.3% of CKD cases in the study population.

**Table 4:** The association between cardiometabolic phenotypes at baseline and incident CKD

Subgroup	cardiometabolic phenotypes	Crude OR (CI)	Adjusted OR (CI)
Total			
	RHC	(Reference)	(Reference)
	MetS	1.67 (1.35–2.07)	1.54 (1.22–1.93)
	CVD	2.33 (1.87–2.91)	2.04 (1.61–2.57)
Male			
	RHC	(Reference)	(Reference)
	MetS	1.67 (1.23–2.25)	1.54 (1.11–2.11)
	CVD	2.85 (2.09–3.87	2.45 (1.77–3.37)
Female			
	RHC	(Reference)	(Reference)
	MetS	1.75 (1.28–2.37)	1.55 (1.11–2.15)
	CVD	2.07 (1.49–2.87)	1.73 (1.22–2.42)
45–65 years			
	RHC	(Reference)	(Reference)
	MetS	1.75 (1.28–2.37)	1.44 (1.08–1.91)
	CVD	2.07 (1.49–2.87	2.07 (1.50–2.81)
65–80 years			
	RHC	(Reference)	(Reference)
	MetS	1.97 (1.37–2.83)	1.80 (1.21–2.67)
	CVD	2.3 (1.63–3.26)	2.17 (1.50–3.13)
> 80 years			
	RHC	(Reference)	(Reference)
	MetS	0.5 (0.06–4.24)	0.22 (0.00-3.83)
	CVD	1.11 (0.22–5.65	0.97 (0.04–15.62)
Nighttime sleep < 7 h			
	RHC	(Reference)	(Reference)
	MetS	1.74 (1.3–2.32)	1.54 (1.12–2.09)
	CVD	2.31 (1.72–3.1	2.07 (1.52–2.81)
Nighttime sleep 7–9 h			
	RHC	(Reference)	(Reference)
	MetS	1.65 (1.18–2.31)	1.50 (1.04–2.13)
	CVD	2.38 (1.66–3.41)	2.01 (1.36–2.94)
Nighttime sleep > 9 h			
	RHC	(Reference)	(Reference)
	MetS	1.33 (0.49–3.58)	1.65 (0.49–5.11)
	CVD	1.95 (0.67–5.69)	1.68 (0.43–5.63)

The adjusted model was adjusted for sociodemographic factors, lifestyle choices, additional comorbidities, and pertinent blood parameters. In the subgroup analysis, we excluded stratification variables and removed them from the adjusted model

Subgroup analyses were conducted to explore potential variations in the association between phenotypes and CKD risk. Stratification variables were excluded from the adjusted models in these analyses. When stratified by gender, participants in both the MetS and CVD phenotype groups (compared to the RHC group) showed a higher risk of CKD in both males and females after adjusting for potential confounders. Specifically, the OR for the MetS phenotype was 1.54 (1.11–2.11) in males and 1.55 (1.11–2.15) in females. Similarly, the OR for the CVD phenotype was 2.45 (1.77–3.37) in males and 1.73 (1.22–2.42) in females. Notably, while the increased CKD risk for females with MetS was comparable to males, the risk increase for females with CVD was slightly lower. Further analyses examined the association by age group (45–65 and 65–80 years). In the younger age group, the risk of CKD increased by 44% for the MetS phenotype (OR: 1.44, 1.08–1.91) and 107% for the CVD phenotype (OR: 2.07, 1.50–2.81). These associations became more pronounced in the older age group, with an 80% increase for the MetS phenotype (OR: 1.80, 1.21–2.67) and a 117% increase for the CVD phenotype (OR: 2.17, 1.50–3.13). This suggests that the impact of cardiometabolic diseases on CKD risk intensifies with advancing age. However, the association between cardiometabolic phenotypes and CKD was no longer statistically significant in individuals aged 80 and above. This may be because other factors, such as comorbidities and overall health status, play a more significant role in this age group.

Stratified results based on nighttime sleep duration revealed an association between sleep deprivation and increased CKD risk in participants with specific cardiometabolic phenotypes. Compared to individuals with standard sleep duration (7–9 h), those with metabolic syndrome and experiencing sleep deprivation (less than 7 h) had a 54% increased risk of CKD (OR = 1.54, 1.12–2.09). Similarly, participants with the CVD phenotype and sleep deprivation exhibited a 107% increased risk (OR = 2.07, 1.52–2.81). Notably, for individuals with extended sleep duration (more than 9 h), although a risk increase was observed, the association was not statistically significant.

## Discussion

This study, analyzing a large cohort of middle-aged and elderly Chinese participants (2015–2020), identified three distinct cardiometabolic phenotypes: MetS, CVD, and RHC. The analysis revealed a strong association between these phenotypes and the development of CKD. Notably, this association with CKD risk from cardiometabolic phenotypes held true for both genders and persisted up to 80 years of age.

Our study builds upon previous research by demonstrating a significantly increased risk of new-onset CKD in participants with MetS and CVD phenotypes compared to those with the RHC phenotype. This finding aligns with the established theory linking cardiometabolic abnormalities to CKD progression. Prior studies by Khalili et al. [24] have shown that both overweight and metabolically unhealthy conditions independently elevate CKD risk [24]. Similarly, research by Kibria and Zheng identified a strong association between cardiovascular disease risk factors and declining kidney function, particularly in older adults. These studies also observed a greater burden of cardiometabolic challenges in individuals with compromised renal health, suggesting a bi-directional relationship [25, 26]. Notably, Leis et al. found that the risk of postoperative acute kidney injury (AKI) differed based on specific cardiometabolic phenotypes [4]. These findings underscore the importance of identifying distinct phenotypes for accurate disease risk assessment and enabling early intervention strategies. Our study reinforces these prior works and further emphasizes the critical role of precise phenotyping within cardiometabolic syndrome for improving CKD prevention and management strategies.

While no significant differences between the MetS and CVD phenotypes were observed within the same sex group without CKD (Table 2), these distinctions were significant in participants who developed CKD (Table 3; Fig. 6A). This suggests potential pathophysiological differences between these phenotypes and their varying impacts on kidney function [27, 28]. Our analysis of gender differences revealed an interesting trend (Table 4): both males and females with MetS or CVD phenotypes experienced an increased risk of CKD compared to the RHC group, females exhibited a slightly lower risk increase for the CVD phenotype specifically. This observation might be due to a combination of factors, including sex-specific physiological and hormonal differences. Estrogen, for example, has been shown to have protective vascular effects, while testosterone may play a more complex role [29]. Mendelsohn and Karas demonstrated that estrogen promotes vasodilation, improves blood flow, and reduces atherosclerosis formation by interacting directly with receptors in blood vessel walls [30]. In addition to hormonal differences, gender differences in genetics, lifestyle, socioeconomics, and health concerns may also influence CKD risk. Our findings highlight the need for further investigation into how sex influences the connection between cardiometabolic diseases and CKD. Future research could explore the potential benefits of estrogen replacement therapy and lifestyle modifications for reducing CKD risk, ultimately paving the way for personalized treatment strategies.

Our analysis revealed an interesting age-dependent trend in the association between cardiometabolic phenotypes and CKD risk (Fig. 6B). While no significant difference in CKD incidence was observed between the CVD and MetS phenotypes in the 45–65 age group, this distinction became statistically significant in the 65–80 years old group. This suggests that the influence of different cardiometabolic states on kidney function may intensify with age. Logistic regression analysis confirmed this escalating effect. The association between MetS and CKD risk increased by 44% in the 45–65 years old and by 80% in the 65–80 years old. Similarly, the association with CVD increased by 107% and 117% in these respective age groups, highlighting the amplifying effect of cardiometabolic abnormalities on CKD risk as we age [31]. Existing research supports this observation: a high prevalence of CKD in older adults is linked to advanced age, diabetes, and metabolic syndrome [32]. While our study did not directly compare the transitions between cardiometabolic statuses, evidence suggests that individuals initially classified as RHC or MetS may develop the other phenotype over time [33, 34]. These findings imply varying disease trajectories, with metabolic syndrome potentially progressing to cardiovascular disease [35–37]. Overall, these results point to a complex interplay between cardiometabolic abnormalities and CKD development, which appears to change across different life stages. Importantly, we observed no significant differences in CKD incidence among individuals over 80 years old, which might be due to limitations in sample size or statistical power in this age group. Future studies are warranted to explore the connection between cardiometabolic diseases and CKD in elderly populations.

Even among individuals with normal sleep duration, those with MetS and CVD exhibited a higher risk of CKD compared to healthy controls. This finding underscores the significant independent effect of cardiometabolic abnormalities on CKD risk. Prior research has shown that insufficient sleep increases cardiometabolic risk [38] and appropriate sleep duration reduces CKD risk [39]. Our study corroborates these findings, demonstrating a significantly elevated CKD risk under conditions of sleep deprivation, particularly for individuals with CVD. The observed trend towards increased risk with excessive sleep warrants further investigation in future studies.

This study utilized LCA to identify three major phenotypes of cardiometabolic syndrome. This approach highlights the importance of a typological approach in managing this complex disorder in clinical practice. LCA, by naturally grouping variable patterns, reveals the complexity of cardiometabolic interactions and offers valuable insights for personalized care. The first category, the metabolic syndrome phenotype, underscores the importance of early identification and management of individuals with insulin resistance and abnormal lipid metabolism. Metabolic syndrome is a known risk factor for CKD [40]. In the United States, for example, three-quarters of kidney failure cases are attributed to diabetes and hypertension [41, 42]. While the overall prevalence of diabetes-related complications has declined, the incidence of renal failure is on the rise in patients with diabetes [41, 43, 44]. In individuals with MetS, an inflammatory state triggered by insulin resistance accelerates CKD progression by elevating pro-inflammatory adipokines and decreasing anti-inflammatory adipokines in the plasma [45]. In addition, dyslipidemia, caused by abnormal lipid metabolism, further worsens renal microvascular damage by promoting atherosclerosis [46]. Intervention strategies for this group may include dietary and lifestyle modifications, glycemic management, and lipid-lowering therapies to reduce the risk of metabolic disease. The cardiovascular disease phenotype is characterized by hypertension and heart disease. Studies have shown that the prevalence of CKD in patients with heart disease is nearly three times that of the general population [47]. Heart failure (HF), in particular, has been strongly associated with CKD development [48]. Impaired cardiac output, high venous pressures, and activation of the renin-angiotensin-aldosterone system and the sympathetic nervous system exacerbate the risk of CKD [48, 49]。Hypertension is not only a key risk factor for CKD, but also disrupts the renal vascular system through atherosclerosis, potentially leading to persistent hypertension and renal failure [50, 51]. Therefore, management for individuals with a cardiovascular disease phenotype should focus on integrated treatment of hypertension and heart disease, employing pharmacological and lifestyle interventions to reduce the risk of cardiovascular complications. The relatively healthy cardiometabolic phenotype demonstrates that cardiometabolic risk factors do not always lead to high-risk outcomes. Even though such individuals are traditionally considered to have a low risk of cardiometabolic disease, renal health monitoring remains crucial. Dynamic changes in metabolic status [52, 53] suggest a potential risk of transitioning to unhealthy state even in healthy populations [54]. Appropriate interventions to improve some of these statuses and prevent their transformation may help to reduce the incidence of CKD [55]. Therefore, regular assessment of cardiac and renal function, in combination with appropriate interventions, is essential for the prevention of CKD.

Traditionally, cardiovascular and metabolic diseases have been the primary targets for preventive and treatment strategies, with cardiovascular and renal diseases often viewed as distinct health entities. However, accumulating evidence from observational studies and clinical trials highlights a significant overlap between these conditions. Recognizing this complex interplay, the American Heart Association (AHA) recently introduced the concept of cardiovascular-renal-metabolic syndrome ^6^. The AHA recommends a tiered screening approach: frequent CKM risk assessment for individuals with established metabolic risk factors, intermediate-frequency screening for those who are overweight or have a history of gestational diabetes, and less frequent screening for healthy adults. Young adults, according to the AHA guidelines, would undergo traditional risk factor screening every 4–6 years.

This study was based on a 5-year follow-up cohort, initiated with baseline physical examination data collected in 2015. However, some limitations should be acknowledged. First, the study design did not account for all potential variables that might influence the relationship between cardiometabolic syndrome phenotypes and CKD. Specifically, we did not include abdominal obesity or serum uric acid levels as cardiometabolic indicators. The selection of cardiometabolic variables in our latent class model was informed by prior studies conducted in diverse populations, including China, the United States, and the United Kingdom. All variables were defined using internationally recognized clinical thresholds (Supplementary Table 3), to ensure consistency and conceptual comparability across different ethnic and national contexts. BMI was included as the sole indicator of adiposity based on its widespread use and strong correlation with general obesity in large cohorts. However, BMI may not fully capture central adiposity, particularly in Asian and some European populations, where visceral fat can accumulate at lower BMI levels. This limitation may influence phenotype classification and its applicability across populations. Future studies may improve phenotypic precision and cross-population generalizability by incorporating direct measures of abdominal obesity, such as waist circumference, into the LCA framework. Additionally, hyperuricemia, which is increasingly recognized as a marker of metabolic and cardiovascular risk, was not incorporated due to data limitations—an acknowledged shortcoming of our study. We have identified these limitations as important directions for future research. Further research is needed to assess whether these phenotypes and their relationship with CKD can be generalized to other Asian subgroups and international populations. Nonetheless, our findings remain consistent with the American Heart Association’s concept of cardiometabolic syndrome and may aid in identifying high-risk populations and guiding preventive strategies to reduce CKD incidence and related public health burdens.

In summary, our study demonstrates a significant association between distinct cardiometabolic syndrome phenotypes and an increased risk of developing CKD. Notably, individuals classified with MetS and CVD phenotypes exhibited a particularly heightened risk. Furthermore, our findings highlight the influence of gender and age on the strength of this association.

Continuous variables are expressed as mean ± standard deviation, or as median (interquartile range). Categorical variables are expressed as frequency (percent). The small letters (a, b, c) in this table refer to comparisons between groups. There is no statistical difference between groups with the same small letters. With 3 latent classes, there are 3 distinct combinations of two classes; the Bonferroni threshold was 0.05/3 = 0.016 when comparing latent classes with each other. BMI: body mass index; SBP: systolic blood pressure; DBP: diastolic blood pressure; HDL-c: high-density lipoprotein cholesterol; TG: triglyceride; High hsCRP: high-sensitivity C-reactive protein levels > 3 mg/L; WBC: white blood cell count; HGB: hemoglobin concentration; HCT: Hematocrit Mean Corpuscular Volume PLT: platelet; Scr: serum creatinine; BUN: blood urea nitrogen; HbA1c: glycated hemoglobin; TC: total cholesterol; FBG: fasting blood glucose; UA: Uric acid; CysC: Cystatin C; HDL-c: high-density lipoprotein cholesterol; LDL-c: low-density lipoprotein cholesterol; CKD Chronic kidney diseases. IQR, interquartile range.

## Supplementary Information

Supplementary Material 1.

## Data Availability

Online repositories contain the datasets used in this investigation. The names of the repositories and the corresponding accession numbers can be found at https://charls.pku.edu.cn/en/. The datasets generated for this study are available upon request from the corresponding author, and the data is provided within the related file named ‘data.csv’.

## Abbreviations

CKD: Chronic Kidney Disease
CHARLS: China Health and Retirement Longitudinal Study
MetS: Metabolic Syndrome
CVD: Cardiovascular Disease
RHC: Relatively Healthy Cardiometabolic
CMS: Cardiometabolic Syndrome
CKM: Cardiovascular Kidney Metabolic
HDL-C: High-density Lipoprotein Cholesterol
GFR: Glomerular Filtration Rate
LCA: Latent class analysis
BIC: Bayesian Information Criterion
AIC: Akaike Information Criterion
AHA: American Heart Association
