# Supplementary material for: Cardiometabolic risk phenotypes and chronic kidney disease incidence in older adults: a nationwide longitudinal cohort study
Source: BMC Public Health. 2025 Jul 29;25:2581. doi: 10.1186/s12889-025-23868-w (PMC12309190; doi:10.1186/s12889-025-23868-w)
Supplement: Supplementary file 1 — Supplementary Material 1. [file 12889_2025_23868_MOESM1_ESM.docx]

**Supplemental Material Table of Contents**

**Appendix 1. Supplementary Tables**

Supplementary Table 1: The definition of eight cardiometabolic conditions involved in the construction of potential classes

Supplementary Table 2: Adjustment of covariates of the model

Supplementary Table 3: The parameter results of the latent class analysis model

Supplementary Table 4: The probability of individuals in each class

Supplementary Table 5: Population Attributable Risk (PAR) for Incident CKD by Latent Cardiometabolic Phenotype

**Appendix 2. Sensitivity Analyses for Latent Class Analysis**

**Validation #1A**

Table 1. Summary statistics of posterior class membership probability for assigned class

Figure 1. Predicted probability of class membership for the assigned class.

Figure 2. Stacked bar chart of individual-level predicted probabilities of class membership

Figure 3. Percentage of cases with a cardiometabolic condition given class membership for the three-class latent class model.

**Validation #1B**

Table 2. Summary statistics of posterior class membership probability for assigned class

Table 3. Crosstabs comparing longitudinal dataset latent class assignments (rows) to cross-sectional dataset latent class assignments (columns)

Figure 5. Percentage of cases with a cardiometabolic condition given class membership for the three-class latent class model.

**Validation #2A**

Table 4. Summary statistics of posterior class membership probability for assigned class

Table 5. Crosstabs comparing derivation cohort latent class assignments (rows) to Full Analytic Cohort latent class assignments (columns)

Figure 6. Percentage of cases with a cardiometabolic condition given class membership for the three-class latent class model.

Figure 7. Predicted probability of class membership for the assigned class – derivation only cohort.

**Validation #2B**

Table 6. Checking posterior class membership probability for assigned class

Table 7. Crosstabs comparing validation cohort latent class assignments (rows) to full analytic cohort latent class assignments (columns)

Figure 8. Percentage of cases with a cardiometabolic condition given class membership for the three-class latent class model.

Figure 9. Predicted probability of class membership for the assigned class – validation only cohort.

**Validation #3**

Table 8. Checking posterior class membership probability for assigned class

Table 9. Crosstabs comparing validation cohort latent class assignments (rows) to Full Analytic Cohort latent class assignments (columns)

Figure 11. Predicted probability of class membership for the assigned class – derivation cohort coefficients in validation cohort data.

**Appendix 1. Supplementary Tables**

**Supplementary** **Table 1. The definition of eight cardiometabolic conditions involved in the construction of potential classes**

The construction of latent classes involved the consideration of eight cardiometabolic conditions: obesity, reduced plasma HDL-C, elevated plasma TG, hypertension, diabetes, arthritis or rheumatism, systemic inflammatory conditions (indicated by raised C-reactive protein), and heart disease. Each of the eight cardiometabolic indicators were considered as separate binary variables based on the presence or absence of an abnormal indicator.

| **Cardiometabolic conditions** | **Definition** |
| --- | --- |
| Obesity | Participants were categorized into BMI groups according to Chinese guidelines: normal weight (BMI 18.5–23.9 kg/m2), overweight (BMI 24.0–27.9 kg/m2), and obesety (BMI ≥28 kg/m2)^2^. |
| Reduced plasma high-density lipoprotein cholesterol (HDL-C) | HDL-C <1.0 mmol/L for men and <1.3 mmol/L for women) or using lipid-lowing drug |
| Elevated plasma triglyceride (TG) | TG ≥1.7 mmol/L or using lipid-lowing drugs^3^. |
| Hypertension | Systolic blood pressure ≥130 mmHg or diastolic blood pressure ≥85 mmHg, self-reported hypertension, or the use of antihypertensive drugs^3^; |
| Diabetes | Self-reported doctor diagnosis of type 2 diabetes, and cases with untreated hyperglycemia and undiagnosed diabetes were detected using HbA1c, with hyperglycemia defined as an HbA1c level ≥48 mmol/mol (6.5%) according to the American Diabetes Association criteria^4^. |
| Systemic inflammation | C-reactive protein (CRP) levels >3 mg/L, according to the American Heart Association and the Center for Disease Control^5^ |
| Heart disease | Self-reported doctor diagnosis of heart attack, coronary heart disease, angina, congestive heart failure, or other heart problems |

**Supplementary** **Table 2. Adjustment of covariates of the model**

This study comprehensively assessed a range of covariates potentially associated with CKD, encompassing sociodemographic factors, lifestyle choices, additional comorbidities, and pertinent blood parameters.

| **covariates** | **Definition** |
| --- | --- |
| Sociodemographic Variables | Age, sex, educational attainment (categorized as elementary school or below, secondary school, and college or above), marital status (married vs. others), and type of residence (rural vs. urban) |
| Lifestyle Variables | Nighttime sleep duration, along with smoking and drinking habits (categorized as yes or no). |
| Additional Comorbidities | Participants were screened for a history of significant comorbid conditions, including but not limited to, cancer, chronic lung diseases, stroke, liver disease, stomach or other digestive diseases, psychiatric conditions, and memory-related disorders. These were based on self-reporting |
| Blood Data | A detailed blood analysis was conducted, measuring a variety of parameters indicative of overall health and specific relevance to CKD. This included the white blood cell count (WBC), hemoglobin concentration (HGB), hematocrit (Hct), mean corpuscular volume (MCV), platelet count (PLT), serum creatinine (SCR), blood urea nitrogen (BUN), uric acid (UA), and cystatin C (CysC). |

**Supplementary** **Table 3. The parameter results of the latent class analysis model**

| N_Class | AIC | BIC | G^2^ | *χ^2^* |
| --- | --- | --- | --- | --- |
| 2 | 61595.38 | 61712.36 | 601.1516 | 643.0767 |
| 3 | 61385.83 | 61564.74 | 373.598 | 379.3323 |
| 4 | 61336.45 | 61577.29 | 306.2236 | 307.6597 |
| 5 | 61291.63 | 61594.4 | 243.4046 | 236.9596 |
| 6 | 61287.01 | 61651.71 | 220.7838 | 214.1062 |
| 7 | 61285.91 | 61712.54 | 201.6781 | 191.347 |
| 8 | 61288.54 | 61777.1 | 186.3108 | 172.9838 |
| 9 | 61291.63 | 61842.12 | 171.4059 | 161.9493 |
| 10 | 61297.25 | 61909.67 | 159.0186 | 147.8659 |

N_Class: the number of latent classes in your Latent Class Analysis (LCA) model; AIC: Akaike Information Criterion; BIC: Bayesian Information Criterion; G2: likelihood ratio test statistic (deviance statistic); *χ^2^*: Pearson's Chi-square test statistic for goodness of fit for a model with 3 latent classes.

**Supplementary** **Table 4:** **The probability of individuals in each class**

| **cardiometabolic conditions** | **MetS** | **CVD** | **RHC** |
| --- | --- | --- | --- |
| Obesity | | | |
| Pr (1) | 0.750244938 | 0.62807057 | 0.260762785 |
| Pr (0) | 0.249755062 | 0.37192943 | 0.739237215 |
| Reduced plasma HDL-C | | | |
| Pr (1) | 0.310319075 | 0.046846729 | 0.050100015 |
| Pr (0) | 0.689680925 | 0.953153271 | 0.949899985 |
| Elevated plasma TG | | | |
| Pr (1) | 0.832219937 | 0.274123089 | 0.172952154 |
| Pr (0) | 0.167780063 | 0.725876911 | 0.827047846 |
| Hypertension | | | |
| Pr (1) | 0.589383852 | 0.858768841 | 0.259561823 |
| Pr (0) | 0.410616148 | 0.141231159 | 0.740438177 |
| Diabetes | | | |
| Pr (1) | 0.404413634 | 0.324891515 | 0.098353007 |
| Pr (0) | 0.595586366 | 0.675108485 | 0.901646993 |
| High hsCRP | | | |
| Pr (1) | 0.36631286 | 0.165806202 | 0.087044895 |
| Pr (0) | 0.63368714 | 0.834193798 | 0.912955105 |
| Heart Disease | | | |
| Pr (1) | 0.178219629 | 0.390681522 | 0.071669769 |
| Pr (0) | 0.821780371 | 0.609318478 | 0.928330231 |
| Arthritis or Rheumatism | | | |
| Pr (1) | 0.408226021 | 0.485089518 | 0.397550908 |
| Pr (0) | 0.591773979 | 0.514910482 | 0.602449092 |

The Latent Class Analysis (LCA) results provided analyze eight cardiometabolic conditions, classifying individuals into three latent classes based on their likelihood of having certain diseases (with 1 indicating the presence of disease and 0 indicating the absence of disease). Below is an analysis of these results, focusing on the conditional probabilities for each health condition across the three classes: Each variable has two possible states, denoted as Pr (1) and Pr (0), representing the probability of individuals in each class having that characteristic.

**Supplementary** **Table 5. Population Attributable Risk (PAR) for Incident CKD by Latent Cardiometabolic Phenotype**

| Latent Class | Proportion in Population | Adjusted OR | Population Attributable Risk (%) |
| --- | --- | --- | --- |
| RHC | 0.663 | 1.00 | 0.0 |
| MetS | 0.199 | 1.54 | 9.7 |
| CVD | 0.138 | 2.04 | 12.6 |

The table summarizes the estimated population attributable risk (PAR) for each latent class, based on adjusted odds ratios (ORs) and population prevalence. The RHC group was used as the reference. PAR values represent the proportion of total CKD incidence attributable to each phenotype, assuming a causal relationship and modifiability of the associated risks.

**Appendix 2. Sensitivity Analyses for Latent Class Analysis**

Visualizations of latent class analyses validations conducted


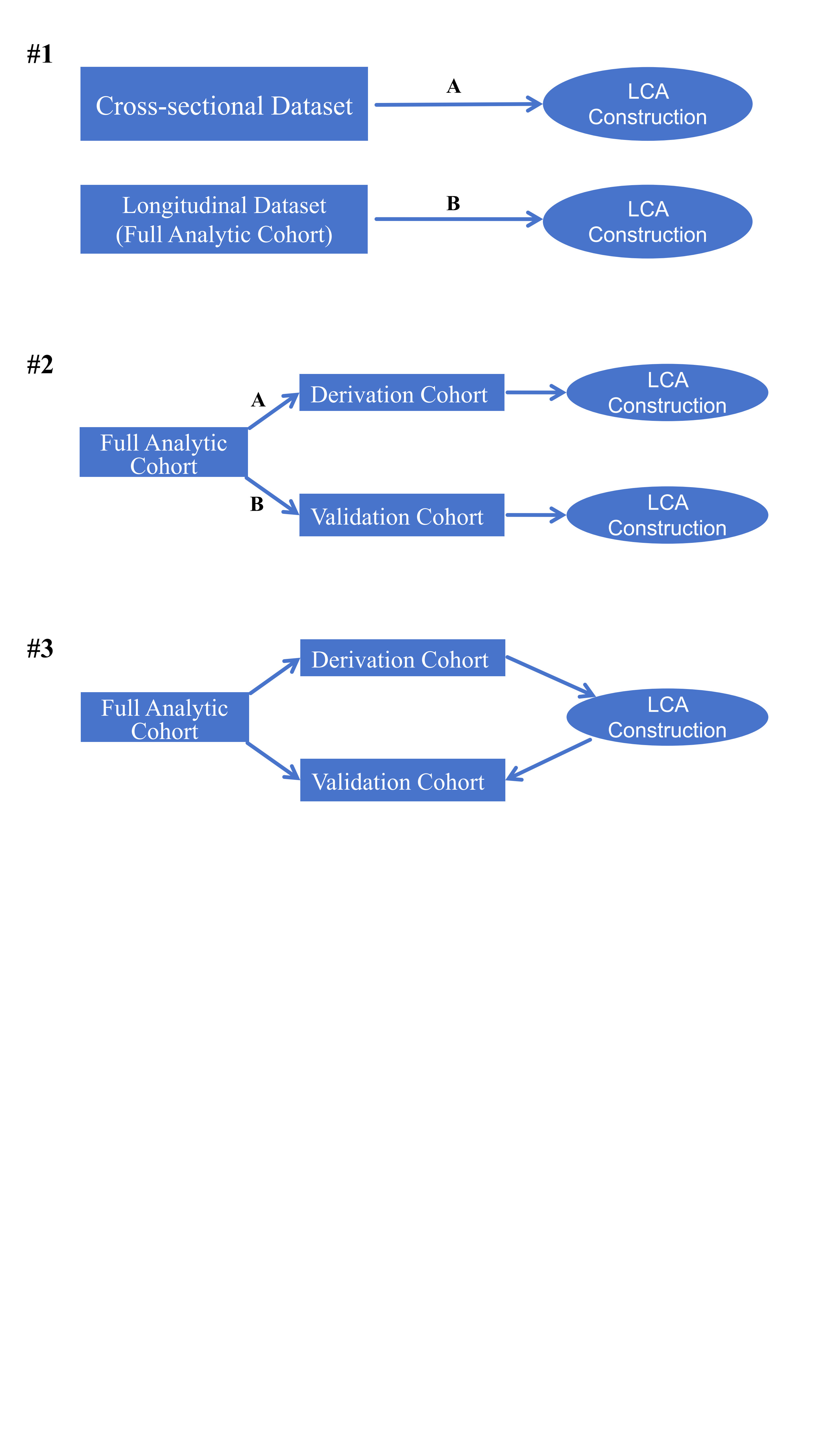


***Validation #1A***

***Latent class analysis (LCA) was run again on the cross-sectional dataset (N=9340) to construct a 3-class model, and the posterior probabilities of class membership for the assigned classes were examined.***

**Table 1** shows the posterior probability of class membership for the assigned latent class, as computed from the cross-sectional dataset LCA construction. As you can see, membership within an assigned class was generally associated with a high predicted probability of membership within the given class. Across the cross-sectional dataset, 14.5% of those in the MetS group had <50% probability of class membership in the given class, 2.1% of those in the CVD group had <50% probability of class membership in the given class, and 3.5 % of those in the RHC group had <50% probability of class membership in that class.

**Figure 1 s**hows the distribution of predicted probability of class membership for the assigned class.

**Figure 2** shows the full probability distributions for everyone assigned to the MetS group (Figure 2a), the CVD group (Figure 2b), and the RHC group (Figure 2c)

**Figure 3** shows percentage of cases with a cardiometabolic condition given class membership for the three-class latent class model.

**Table 1. Summary statistics of posterior class membership probability for assigned class**

|  | Mean | St. Dev | Maximum | Minimum |
| --- | --- | --- | --- | --- |
| MetS | 0.675 | 0.158 | 0.986 | 0.356 |
| CVD | 0.756 | 0.169 | 0.992 | 0.406 |
| RHC | 0.857 | 0.156 | 0.986 | 0.41 |

Of the 1,768 people assigned MetS, 257 (14.5 %) had <50% probability of class membership

Of the 1596 people assigned CVD, 33 (2.1%) had <50% probability of class membership

Of the 5,976 people assigned RHC, 207 (3.5 %) had <50% probability of class membership


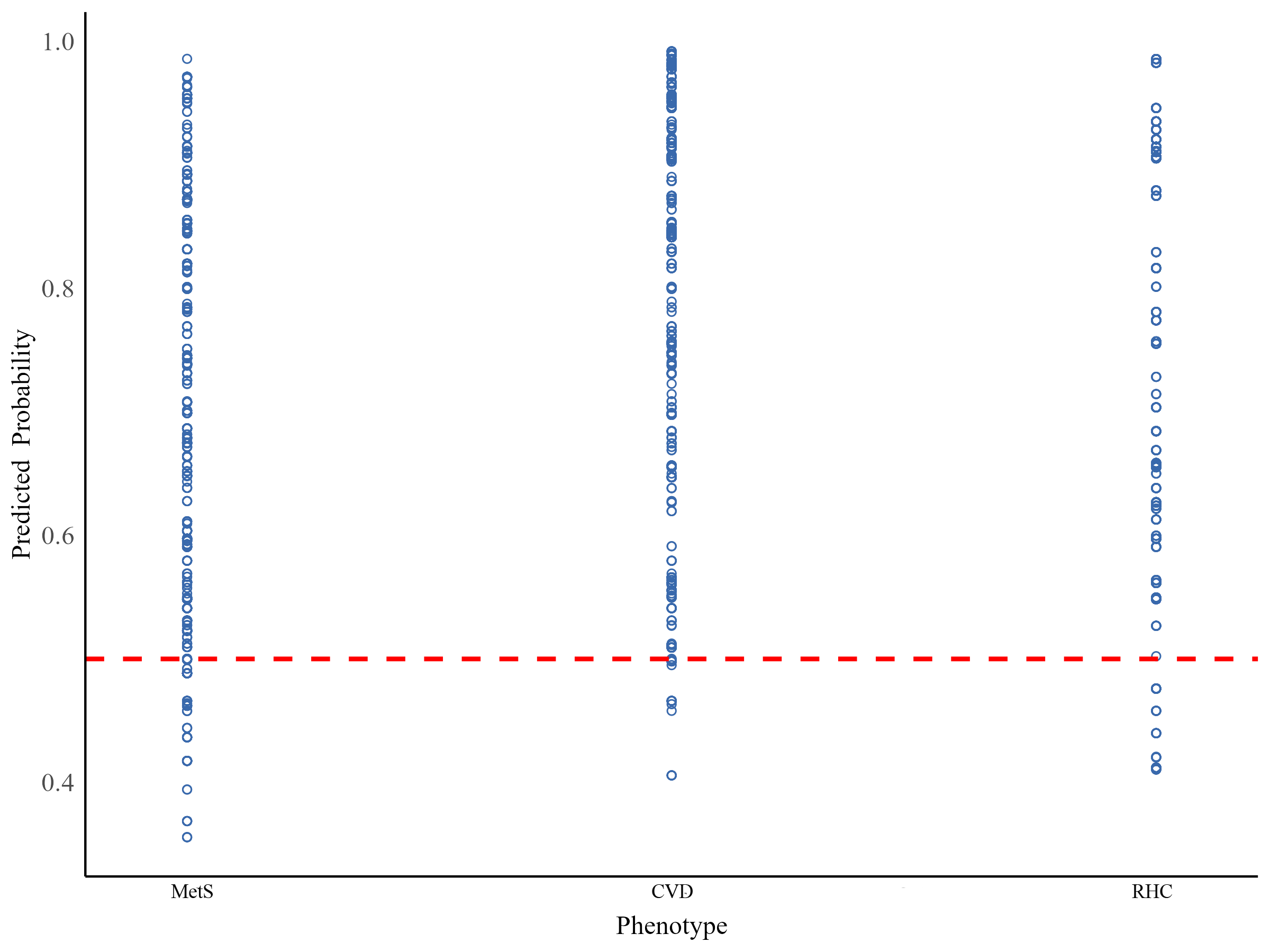


**Figure 1. Predicted probability of class membership for the assigned class.**

**
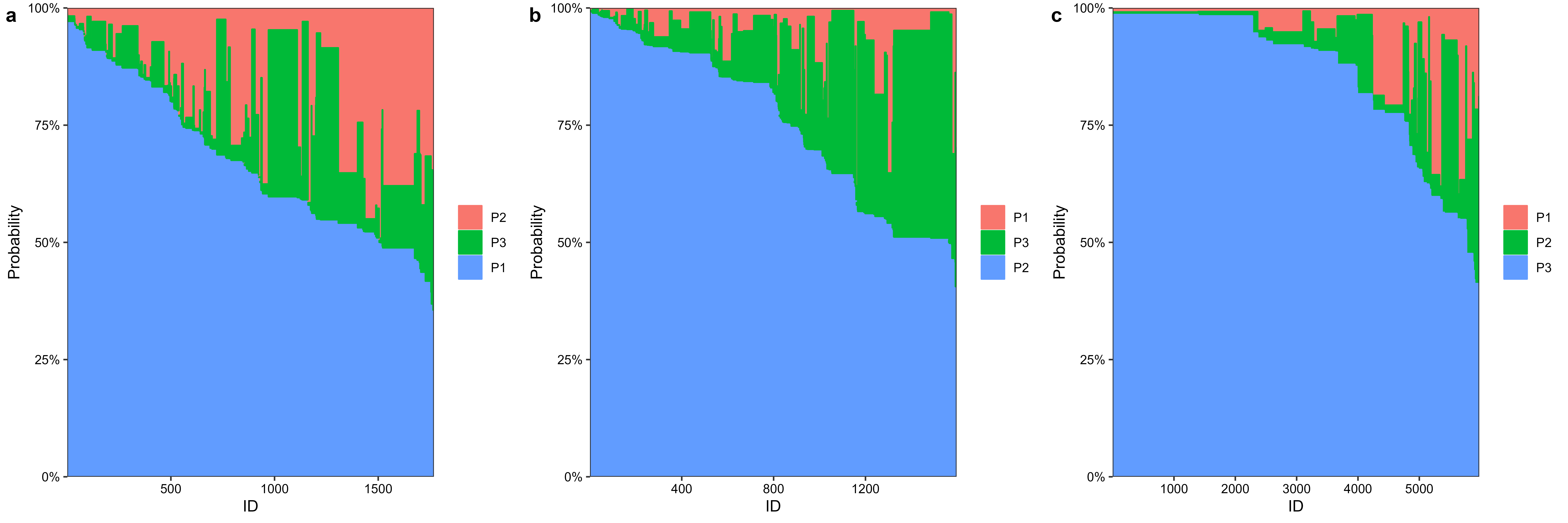
**

**Figure 2. Stacked bar chart of individual-level predicted probabilities of class membership**


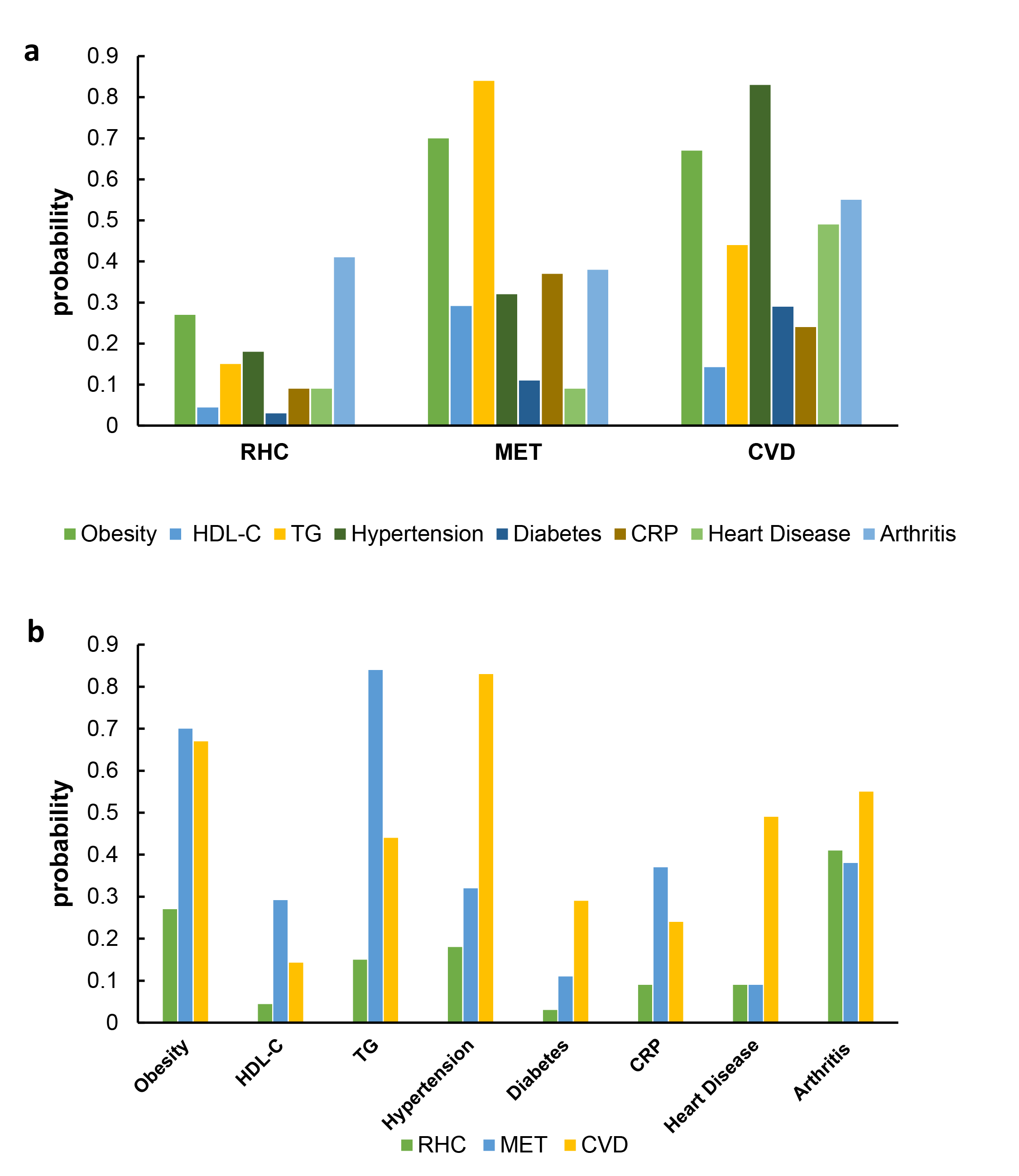


**Figure 3. Percentage of cases with a cardiometabolic condition given class membership for the three-class latent class model.**

**Validation #1B**

***Latent class construction was run for the 3-class model within only those in the longitudinal dataset (N=7195). Posterior probability of class membership was then compared with that from the latent classes constructed within the cross-sectional dataset (N=9340).***

**Table 2** shows the posterior probability of class membership for the assigned latent class, as computed from the full data cohort LCA construction. Similar classes emerged to the longitudinal dataset (**Figure 5**). As you can see, membership within an assigned class was generally associated with a high predicted probability of membership within the given class.

Across the longitudinal dataset, 0.5% of those in the RHC group had <50% probability of class membership in the given class, 7.0% of those in the MetS group had <50% probability of class membership in the given class, and 10.1 % of those in the CVD group had <50% probability of class membership in that class.

**Figure 4** shows the distribution of predicted probability of class membership for the assigned class.

**Table 2. Summary statistics of posterior class membership probability for assigned class**

|  | Mean | St. Dev | Maximum | Minimum |
| --- | --- | --- | --- | --- |
| MetS | 0.733 | 0.169 | 0.993 | 0.366 |
| CVD | 0.651 | 0.141 | 0.925 | 0.347 |
| RHC | 0.835 | 0.166 | 0.986 | 0.393 |

Of the 1,433 people assigned MetS, 101 (7.0 %) had <50% probability of class membership

Of the 993 people assigned CVD, 101 (10.1 %) had <50% probability of class membership

Of the 4,769 people assigned RHC 23 (0.5 %) had <50% probability of class membership

**Table 3. Crosstabs comparing longitudinal dataset latent class assignments (rows) to cross-sectional dataset latent class assignments (columns)**

|  |  | cross-sectional dataset | | |
| --- | --- | --- | --- | --- |
|  |  | MetS | CVD | RHC |
| longitudinal  dataset | MetS | 1381(97.25) | 0(0.00) | 52(1.09) |
|  | CVD | 6(0.42) | 987(96.58) | 0(0.00) |
|  | RHC | 33(2.32) | 35(3.42) | 4701(98.91) |

Data are presented as n (column %).


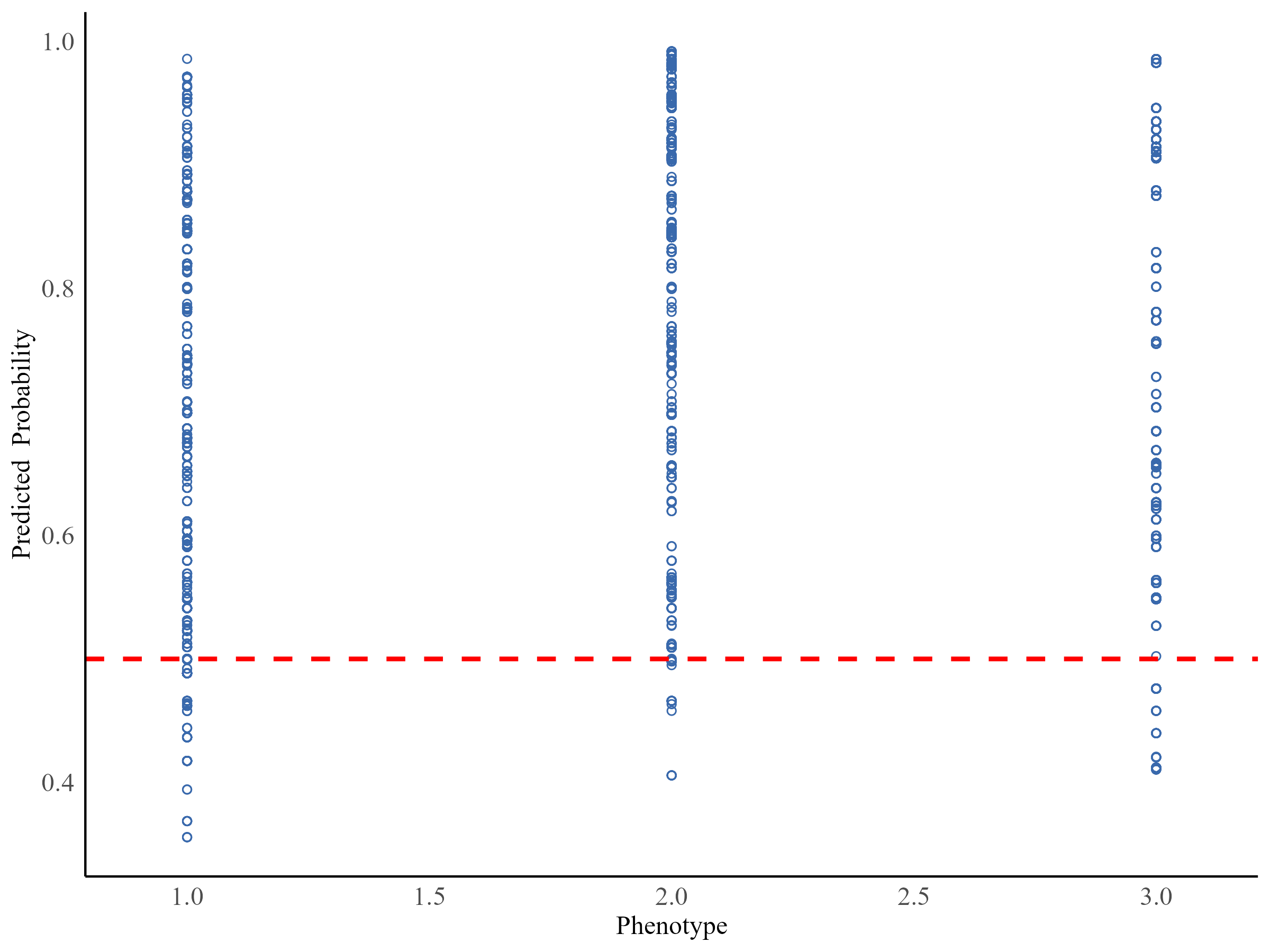


**Figure 4. Predicted probability of class membership for the assigned class.**


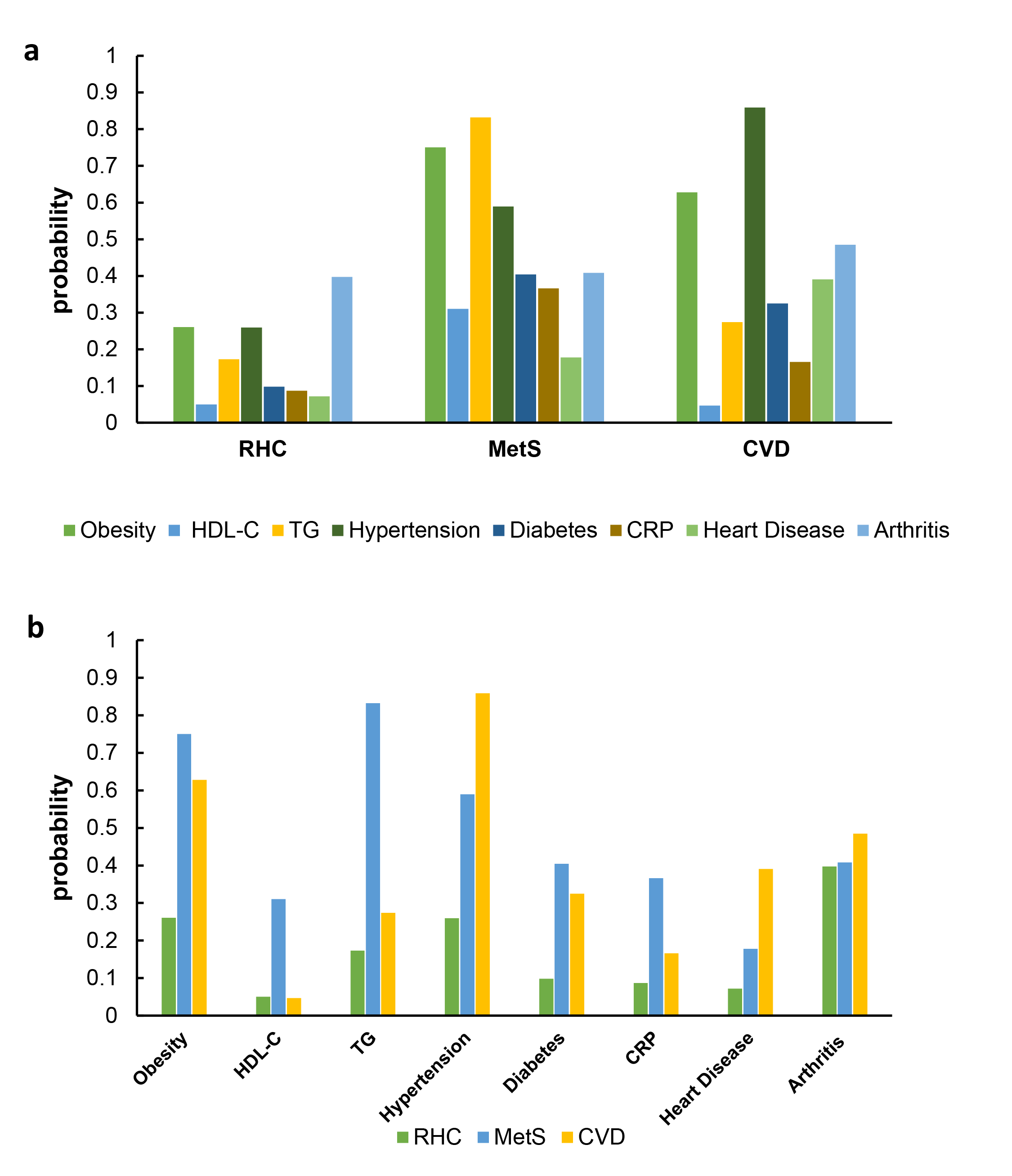


**Figure 5. Percentage of cases with a cardiometabolic condition given class membership for the three-class latent class model.**

**Validation #2A**

***Latent class construction was run again for the 3-class model within only those in the derivation cohort (N* = 5,036*). Posterior probability of class membership was then compared with that from the latent classes constructed within the full analytic cohort.***

**Table 4** below shows the posterior probability of class membership for the assigned latent class, as computed from a new LCA model constructed only within the derivation cohort. Similar classes emerged to the full cohort, as would be expected given the derivation cohort makes up a significant portion of the full analytic cohort **(Figure 6**). As you can see, membership within an assigned class was generally associated with a high predicted probability of membership within the given class (**Figure7, Table 4**). Across the derivation cohort, 2.6% of those in the CVD group had <50% probability of class membership in the given class, 5.1% of those in the MetS group had <50% probability of class membership in the given class, and 4.2% of those in the RHC group had <50% probability of class membership in that class.

**Table 5** shows high agreement of LCA models constructed in the derivation cohort with those classes constructed in the full cohort for those in the RHC group and those in the MetS group.

**Table 4. Summary statistics of posterior class membership probability for assigned class**

| Group | Mean | StDev | Min | Max |
| --- | --- | --- | --- | --- |
| CVD | 0.64 | 0.137 | 0.419 | 0.916 |
| MetS | 0.734 | 0.17 | 0.402 | 0.995 |
| RHC | 0.84 | 0.17 | 0.403 | 0.988 |

Of the 647 people assigned CVD, 17 (2.6%) had <50% probability of class membership

Of the 1146 people assigned MetS, 59 (5.1%) had <50% probability of class membership

Of the 3243 people assigned RHC, 137 (4.2%) had <50% probability of class membership

**Table 5.** **Crosstabs comparing derivation cohort latent class assignments (rows) to Full Analytic Cohort latent class assignments (columns)**

|  |  | Full Analytic Cohort | | |
| --- | --- | --- | --- | --- |
|  |  | MetS | CVD | RHC |
| Derivation Cohort | MetS | 1052 (100.0%) | 4 (0.58%) | 90 (2.73%) |
|  | CVD | 0 (0.0%) | 647 (93.77%) | 0 (0.0%) |
|  | RHC | 0 (0.0%) | 39 (5.65%) | 3204 (97.27%) |

Data are presented as n (column %).


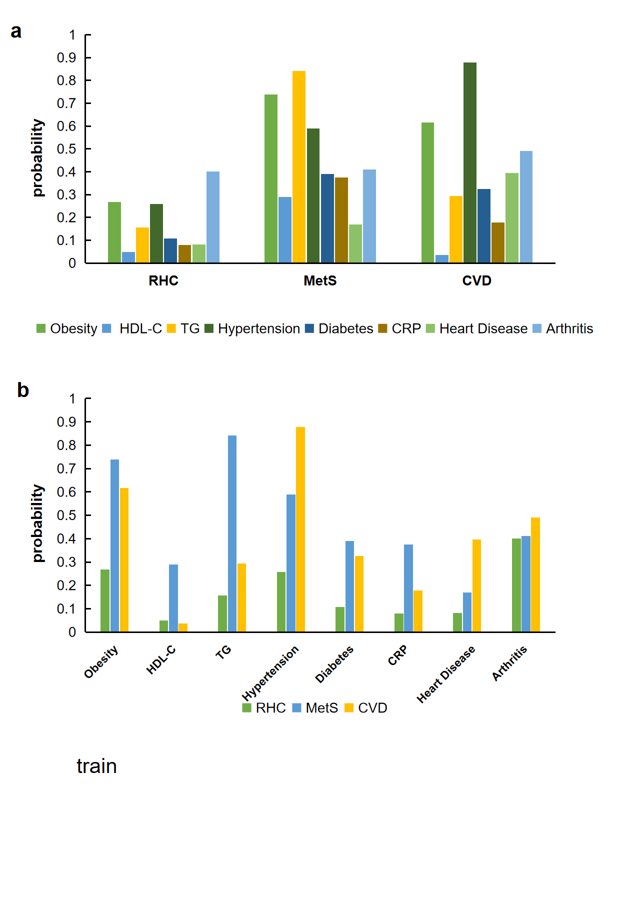


**Figure 6. Percentage of cases with a cardiometabolic condition given class membership for the three-class latent class model.**


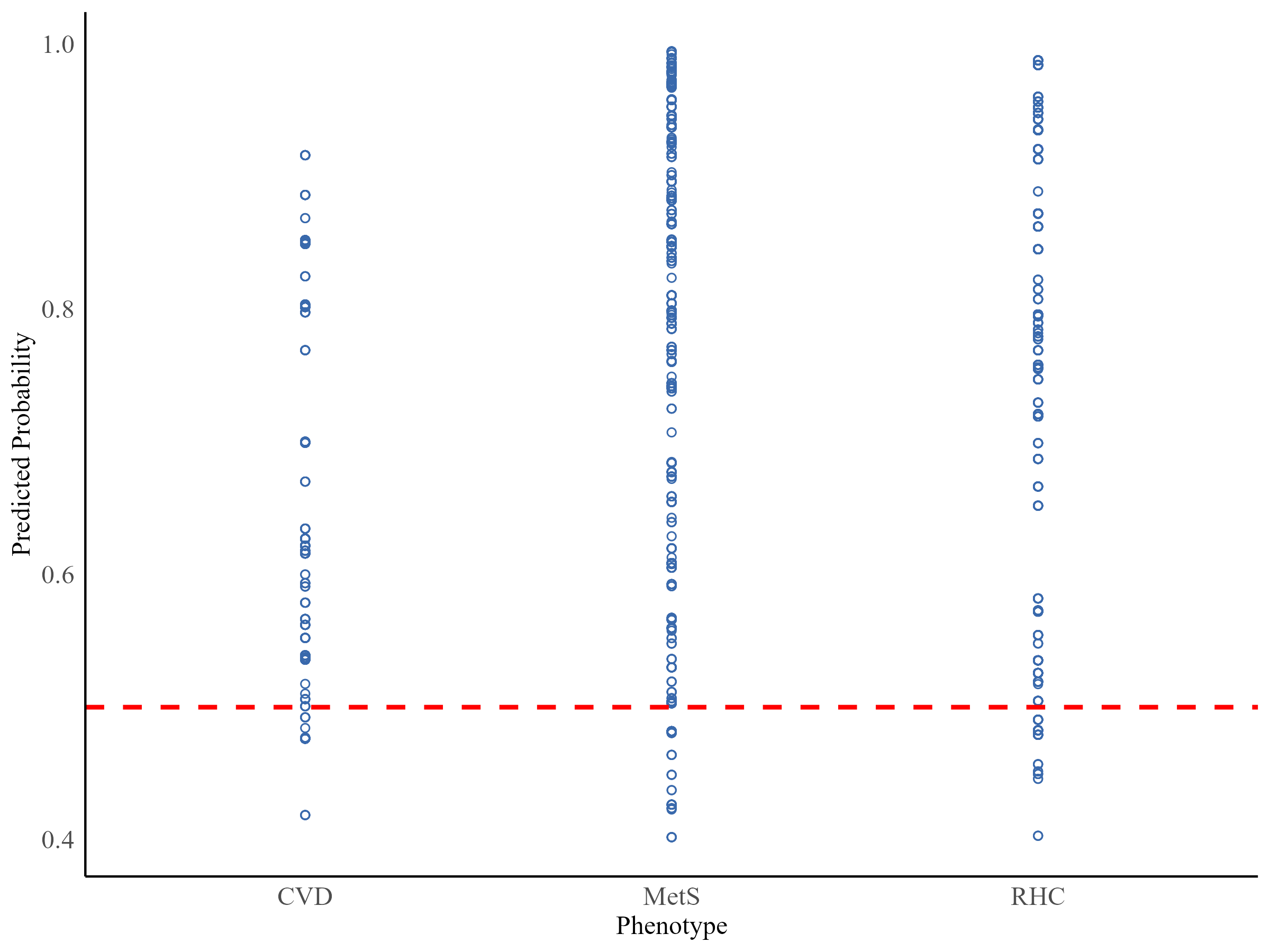


**Figure 7. Predicted probability of class membership for the assigned class – derivation only cohort.**

**Validation #2B**

***Latent class construction was run again for the 3-class model within only those in the validation cohort (N=*2,159*). Posterior probability of class membership was then compared with that from the latent classes constructed within the full analytic cohort.***

**Table 6** below shows the posterior probability of class membership for the assigned latent class, as computed from a new LCA model constructed only within the validation cohort. Similar classes emerged in the validation cohort, though the CVD group was characterized by a markedly higher probability of heart disease, despite similarly high obesity levels in both the MetS and CVD groups (**Figure 8**). Membership within an assigned class was generally associated with a high predicted probability of membership within the given class (**Figure 9, Table 6**). Across the validation cohort, 6.7% of those in the MetS group had <50% probability of class membership in the given class, 0.8% of those in the RHC group had <50% probability of class membership in the given class, and 4.2% of those in the CVD group had <50% probability of class membership in that class.

**Table 7** shows high agreement of LCA models constructed in the validation cohort with those classes constructed in the full cohort.

**Table 6. Checking posterior class membership probability for assigned class**

| Group | Mean | StDev | Min | Max |
| --- | --- | --- | --- | --- |
| RHC | 0.855 | 0.127 | 0.427 | 0.977 |
| CVD | 0.652 | 0.14 | 0.407 | 0.919 |
| MetS | 0.729 | 0.178 | 0.439 | 0.991 |

Of the 1343 people assigned RHC, 11 (0.8%) had <50% probability of class membership

Of the 447 people assigned CVD, 19 (4.2%) had <50% probability of class membership

Of the 369 people assigned MetS, 25 (6.7%) had <50% probability of class membership

**Table 7. Crosstabs comparing validation cohort latent class assignments (rows) to full analytic cohort latent class assignments (columns)**

|  |  | Full Analytic Cohort | | |
| --- | --- | --- | --- | --- |
|  |  | MetS | CVD | RHC |
| Validation Cohort | MetS | 362 (95.0%) | 7 (2.3%) | 0 (0.0%) |
|  | CVD | 13 (3.4%) | 296 (97.7%) | 138 (9.4%) |
|  | RHC | 6 (1.6%) | 0 (0.0%) | 1337 (90.6%) |

**
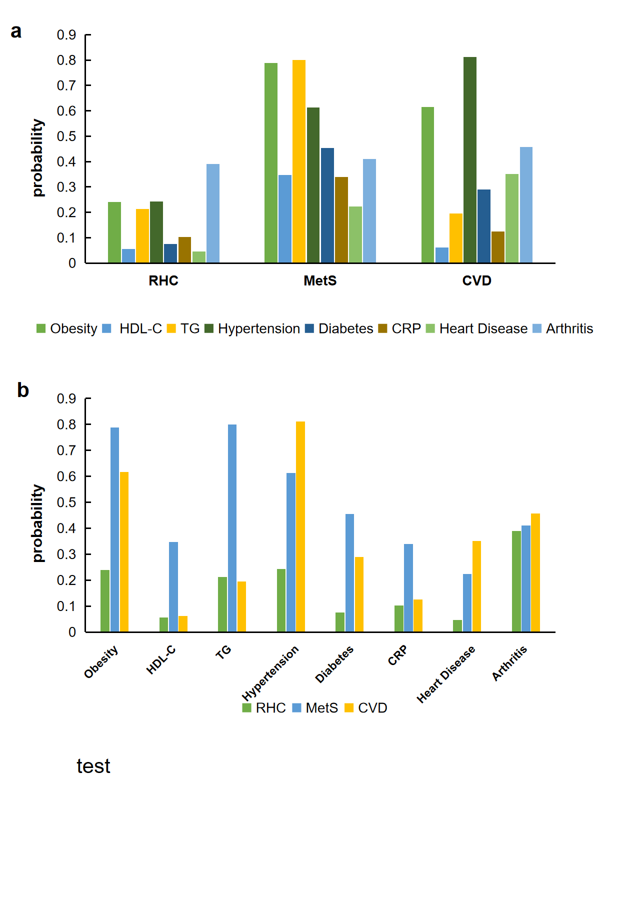
**

**Figure 8. Percentage of cases with a cardiometabolic condition given class membership for the three-class latent class model.**


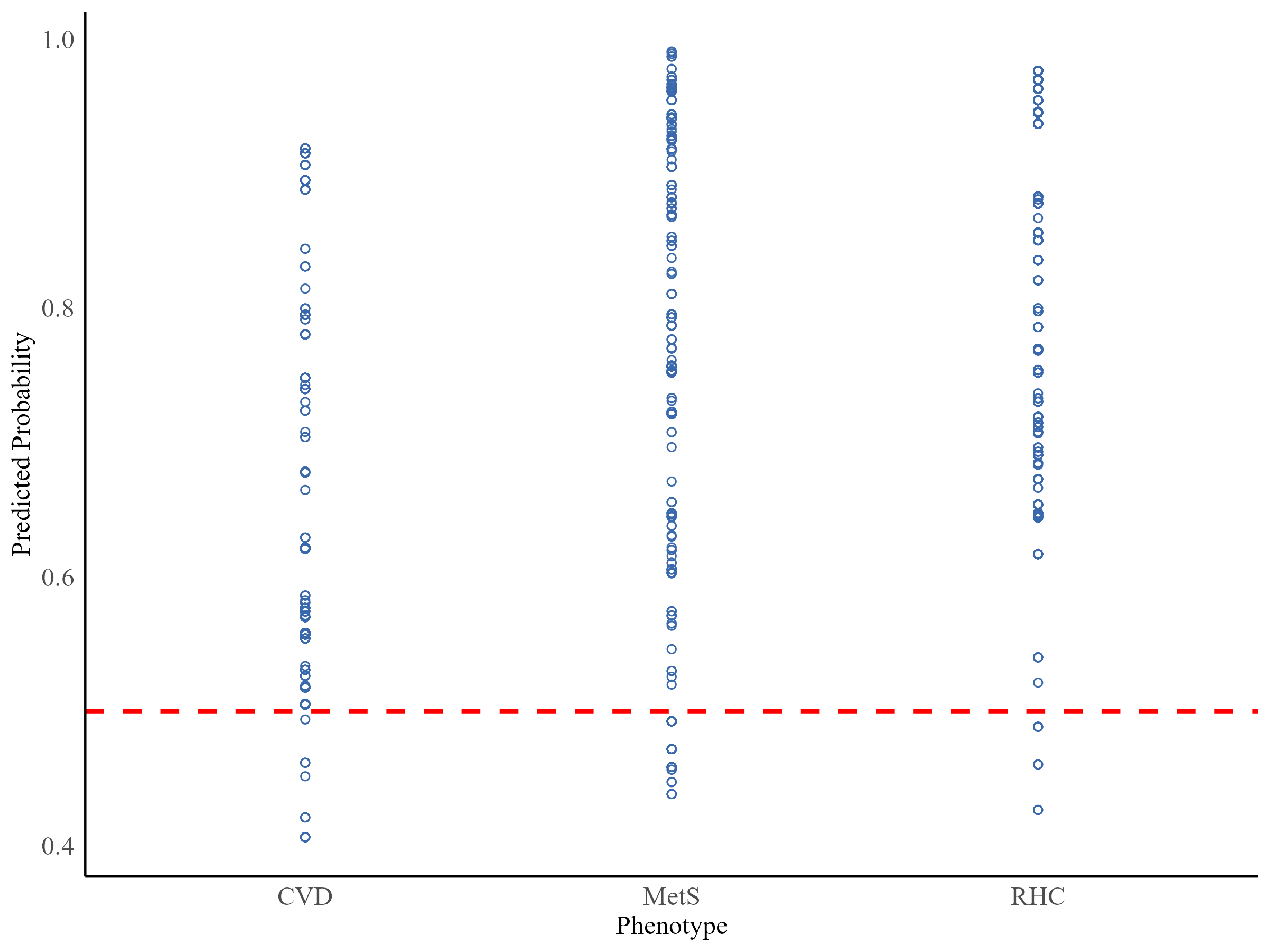


**Figure 9. Predicted probability of class membership for the assigned class – validation only cohort.**

**Validation #3**

***Latent class construction was run for the 3-class model within only those in the derivation cohort. Model coefficients were then hand-coded into those in the validation cohort, and the latent class with the highest posterior predicted value was assigned for class membership. Posterior probability of class membership was then compared with that from the latent classes constructed within the full analytic cohort.***

There were generally high predicted probabilities of class membership when the coefficients from the derivation only LCA model were applied to the validation cohort (**Table 8, Figure 11**). Across the validation cohort, 8.6% of those in the MetS group had <50% probability of class membership in the given class, 3.6% of those in the RHC group had <50% probability of class membership in the given class, and 2.8% of those in the CVD group had <50% probability of class membership in that class.

**Table 9** shows high agreement of LCA models constructed in the validation cohort with those classes constructed in the full cohort.

**Table 8. Checking posterior class membership probability for assigned class**

| Group | Mean | StDev | Min | Max |
| --- | --- | --- | --- | --- |
| RHC | 0.798 | 0.166 | 0.407 | 0.963 |
| CVD | 0.72 | 0.125 | 0.434 | 0.95 |
| MetS | 0.713 | 0.172 | 0.373 | 0.996 |

Of the 1157 people assigned RHC, 42 (3.6%) had <50% probability of class membership

Of the 456 people assigned CVD, 13 (2.8%) had <50% probability of class membership

Of the 546 people assigned MetS, 47 (8.6%) had <50% probability of class membership

**Table 9. Crosstabs comparing validation cohort latent class assignments (rows) to** **Full Analytic Cohort latent class assignments (columns)**

|  |  | Full Analytic Cohort | | |
| --- | --- | --- | --- | --- |
|  |  | MetS | CVD | RHC |
| Validation Cohort | MetS | 375 (98.33 %) | 0 (0.00 %) | 171 (11.61 %) |
|  | CVD | 6 (1.57 %) | 303 (100.00 %) | 147 (9.97 %) |
|  | RHC | 0 (0.00 %) | 0 (0.00 %) | 1157 (78.41 %) |


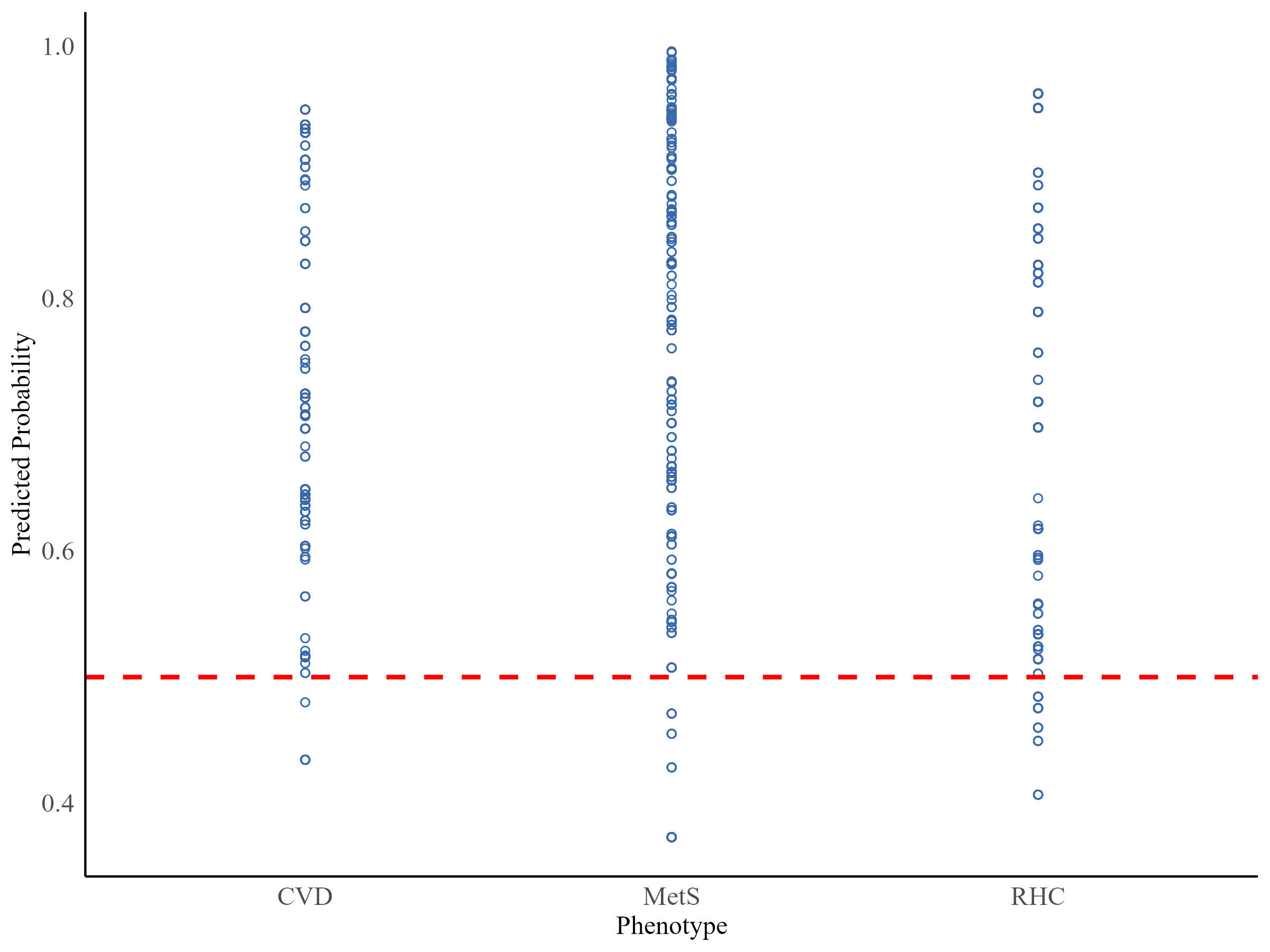


**Figure 11. Predicted probability of class membership for the assigned class – derivation cohort coefficients in** **validation cohort data.**
